# Electrospinning Versus Chemical Polymerization for Conductive Polymer Fibers: Reproducibility, Electrical Performance and Wearable Sensing

**DOI:** 10.3390/polym18212730

**Published:** 2026-11-03

**Authors:** Abdullah Bin Bashir, Dilip Depan

**Affiliations:** Chemical Engineering Department, Institute for Materials Research and Innovation, University of Louisiana at Lafayette, P.O. Box 44130, Lafayette, LA 70504-4130, USA; abdullah-ahmed.bashir1@louisiana.edu

**Keywords:** conductive polymers (CPs), polyaniline (PANI), polypyrrole (PPy), PEDOT:PSS, electrospinning, chemical polymerization, nanofibers, smart textiles, morphology control, energy storage devices

## Abstract

Conductive polymers (CPs) such as polyaniline (PANI), polypyrrole (PPy) and poly(3,4-ethylenedioxythiophene) (PEDOT) combine electronic function with the processability of plastics, and one-dimensional CP fibers add high surface area and directional charge transport. This review compares chemical oxidative polymerization and electrospinning against three criteria: how fiber geometry is reported and controlled, electrical performance, and wearable-sensor behavior. The comparison separates the chemistry that forms the polymer from the operation that forms the fiber, because the two are frequently conflated. Fiber diameter is reported as a numeric range wherever a dedicated spinning step defines it, whether by electrospinning or by wet spinning, and qualitatively where the fiber forms by precipitation during polymerization. This is a difference in reporting practice and in dimensional uniformity; it is not evidence of batch to batch or inter-laboratory reproducibility, for which no data were found for either route. Electrical comparison is complicated by measurement geometry, since single-fiber, mat, pellet and sheet-resistance values span one to three orders of magnitude across the surveyed literature and are not interchangeable. Where a single study varies one factor at a time, carrier removal and post-spinning conversion recover much of the apparent electrical deficit of electrospun fibers. Reported wearable-sensing metrics are not comparable across transduction modes, and the surveyed devices differ in composition and added conductive phases, so no ranking by fabrication route is drawn. The two routes are complementary: chemical polymerization supplies the conducting phase and scales by batch volume, while electrospinning sets the fiber architecture.

## 1. Introduction

Conductive polymers (CPs) are π-conjugated organic materials whose electrical conductivity can be tuned across many orders of magnitude by chemical or electrochemical doping. Since the discovery that iodine-doped polyacetylene becomes metallic [1], polyaniline (PANI), polypyrrole (PPy), polythiophene and poly(3,4-ethylenedioxythiophene) (PEDOT) have been studied intensively [2,3]. Doping introduces charge-bearing polarons and bipolarons along the conjugated backbone, shifting these materials from semiconducting toward metallic behavior and allowing conductivity to be switched reversibly by protonation or redox cycling [3,4]. Their appeal lies in a combination of benefits that a few materials offer: metal-like charge transport together with the low cost, low density and solution processability of plastics [5,6,7]. Production is no longer a laboratory curiosity, as PEDOT:PSS dispersions are manufactured at industrial scale by major commercial suppliers, with Heraeus Clevios™ formulations widely used in antistatic coatings, OLEDs and solar cells [3]. Because CPs are also biocompatible and readily surface-modified, they have been adopted in biosensors, tissue scaffolds, fuel cells, energy storage and wearable electronics [8,9,10].

Shaping these polymers into one-dimensional fibers adds advantages that films and powders cannot provide. Fibers combine a high specific surface area with mechanical compliance and continuous, directional charge-transport pathways, which together favors rapid charge transfer, efficient ion access and tolerance of bending or stretching [8,11,12,13]. These are precisely the requirements of electronic textiles, wearable sensors, actuators and fiber-shaped energy storage devices, where the active material must survive repeated deformation while remaining in electrical contact [14,15,16]. Fibrous mats also offer tunable porosity, which governs electrolyte penetration in electrodes and analyte access in sensors [17,18,19]. Two fabrication strategies dominate the literature: chemical (oxidative) polymerization and electrospinning.

Chemical polymerization proceeds by oxidative coupling of a monomer such as aniline, pyrrole or 3,4-ethylenedioxythiophene (EDOT) in the presence of an oxidant, typically ammonium persulfate (APS) or FeCl_3_, together with an acid dopant [3,20]. It is attractive because it is simple and inexpensive, requires no equipment beyond stirring and cooling, and scales by increasing batch volume [10,21]. Doping is built into the synthesis, so strong acids introduced during polymerization yield highly conducting products directly [20,22]. Its weakness is morphological: conventional solution polymerization gives powders, granular deposits or thick films rather than defined fibers, and fiber-like architectures must be coaxed out of it through interfacial polymerization, template-assisted growth, in situ deposition onto an existing textile, or wet spinning of a doped polymer solution [11,16,23,24]. In situ coating of cotton, nylon or pre-spun mats is widely used and gives good adhesion, enabling the resulting textiles to function as shielding layers, strain sensors and electrochemical electrodes, though their sheet resistance depends on coating thickness and is reported inconsistently in the literature [25,26]. Neat CPs are also brittle and largely insoluble, which limits post-processing [13,21].

Electrospinning takes the opposite approach. Rather than growing the polymer into a shape, it draws an already-formed polymer solution into a fiber using an electric field, producing continuous filaments from tens of nanometers to a few micrometres in diameter [27,28]. The technique is inexpensive, compatible with a wide range of polymers, and gives direct control over fiber diameter, alignment and mat porosity through solution properties, applied voltage and collector design [4,18,29]. The resulting nonwoven mats have exceptional surface-to-volume ratios and are easy to handle and integrate into devices [12,30,31,32]. The difficulty is that neat CPs are difficult to electrospun, because their rigid, low-molecular-weight chains do not entangle sufficiently and the jet breaks into beads or fragments instead of continuous fibers [4,33,34]. Practical routes therefore rely on blending with a carrier polymer such as poly(ethylene oxide) (PEO), poly(vinylpyrrolidone) (PVP) or polyacrylonitrile (PAN), on coaxial spinning with a sacrificial shell, or on spinning an inert template that is subsequently converted by in situ or vapor-phase polymerization [4,35,36]. Each of these restores spinnability at some cost in conductivity, throughput or additional processing steps [4,37,38].

**Figure 1:**
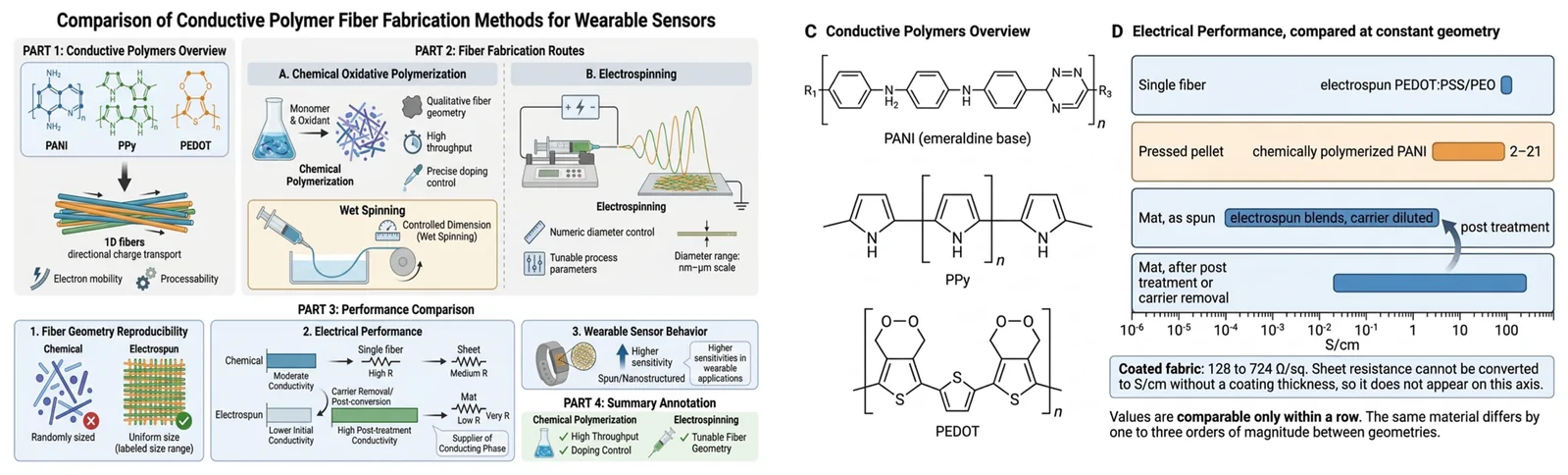
Comparison of the two dominant routes to conductive polymer fibers. In chemical (oxidative) polymerization, the polymer is grown into its final morphology, which is set indirectly by the reaction route; in electrospinning, the morphology is imposed on an already-formed polymer by the electric field and collector geometry.

These differences are not symmetrical, and Figure 1 summarizes why. In chemical polymerization, the fiber is a by-product of the reaction: geometry emerges indirectly from monomer concentration, acidity, oxidant ratio and mixing regime, and it is therefore difficult to specify in advance or reproduce across laboratories. In electrospinning, the geometry is imposed by a dedicated fiber-forming step and is set by parameters the operator controls directly. The position taken in this review is that architecture is best assigned to a spinning process and chemistry is best assigned to supplying the conducting phase. The strongest mechanical result in the whole surveyed set supports this reading rather than contradicting it: the ultrafine PANI of Fang et al. was produced by wet spinning, that is, by extrusion, coagulation and drawing, and not by precipitation from a polymerization bath [33]. Where the two are combined, with an electrospun scaffold converted afterwards by oxidative or vapor-phase polymerization, the resulting fibers recover much of the conductivity that a diluting carrier would otherwise cost [16,24,36].

The reviews cited above survey electrospun conductive polymers [4], polypyrrole nanofibers [11], PEDOT-based nanofibers [35] and conductive textiles [15,26] within a single route or a single material. What this review adds is not further coverage but three things that a within-route survey cannot provide: an explicit separation of the synthesis, fiber formation, coating and post-treatment stages, so that results are attributed to the stage that produced them; a statement of the measurement geometry attached to every conductivity value compiled, with the conditions recorded alongside it, and an assessment of what the reporting practice of each route does and does not establish about reproducibility.

Four stages are distinguished throughout and are kept separate in the text, the tables and the figures. Synthesis is the chemistry by which the conductive polymer is formed. Fiber formation is the operation by which the fiber geometry is established, whether by electrospinning, wet spinning or precipitation during the polymerization itself. Coating is the deposition of a conducting phase onto a fiber formed from another material. Post-treatment is any step applied after the fiber exists, including annealing, solvent or acid treatment and carrier removal. These stages are independent, and the result produced by one combination is not evidence about another. Where a system combines stages, as in a template fiber coated by vapor-phase polymerization, its performance is attributed to the combination and not to either stage alone.

### Literature Search and Selection

The literature was searched using google scholar between 2015 and 2025 using combinations of the term’s conductive polymer, polyaniline, polypyrrole, PEDOT, nanofiber, electrospinning, chemical oxidative polymerization, wet spinning and wearable sensor. A record was included where it reported a conductive polymer in fiber or fiber-coated form together with at least one of the following, fiber dimension, electrical conductivity or device performance, and it was excluded where the conductive polymer appeared only as a film, powder or bulk solid, or where no original measurement was reported. The nominal window was 2015 to 2025. Earlier records were retained in three defined cases: foundational reports of the polymers and of their electrospinning, for which no later equivalent exists; the original source of a compiled value, where later work only restates it; and reviews cited in Section 1 to establish what has already been surveyed. Compiled entries were extracted from the primary source in every case, and the measurement conditions were taken from the same source or marked as not reported.

## 2. Chemical Polymerization of Conductive Polymers

### 2.1. Basic Mechanism and Chemistry

Chemical oxidative polymerization of conductive polymers proceeds by monomer oxidation and coupling to form conjugated chains. In practice, aniline, pyrrole and 3,4-ethylenedioxythiophene (EDOT) are commonly used monomers. In a typical batch reaction, the monomer is dissolved in a strong acid (e.g., HCl or H_2_SO_4_) and mixed with a stoichiometric oxidant (often ammonium persulfate, APS, or ferric chloride) under cooling [39]. The oxidant converts the monomer to a radical cation, which couples into head-to-tail or head-to-head linkages while the oxidant is consumed stoichiometrically rather than catalytically. The required molar ratio is specific to the monomer: for aniline, the recommended peroxydisulfate-to-aniline ratio is 1.25, and for pyrrole oxidized by ferric chloride, it is higher again. For example, chemical polymerization of aniline in approximately 1M HCl with APS yields emeraldine-salt polyaniline with protonated (doped) imine units [39]. Polypyrrole follows the same chemistry and is typically oxidized by APS or FeCl_3_ in aqueous acid. EDOT polymerization uses FeCl_3_ in organic solvent or iron(III) tosylate rather than APS, because APS has limited stability in the anhydrous or weakly polar conditions required for EDOT. For PANI and PPy, the strongly acidic medium protonates the polymer backbone, creating delocalized polaron and bipolaron states; this acid–base doping is facile and reversible, and PANI’s conductivity can be toggled by protonation/deprotonation [40]. PEDOT is oxidation-doped by the Fe^3+^ oxidant directly, with PSS or tosylate serving as the charge-balancing counterion rather than a protic acid.

**Figure 2:**
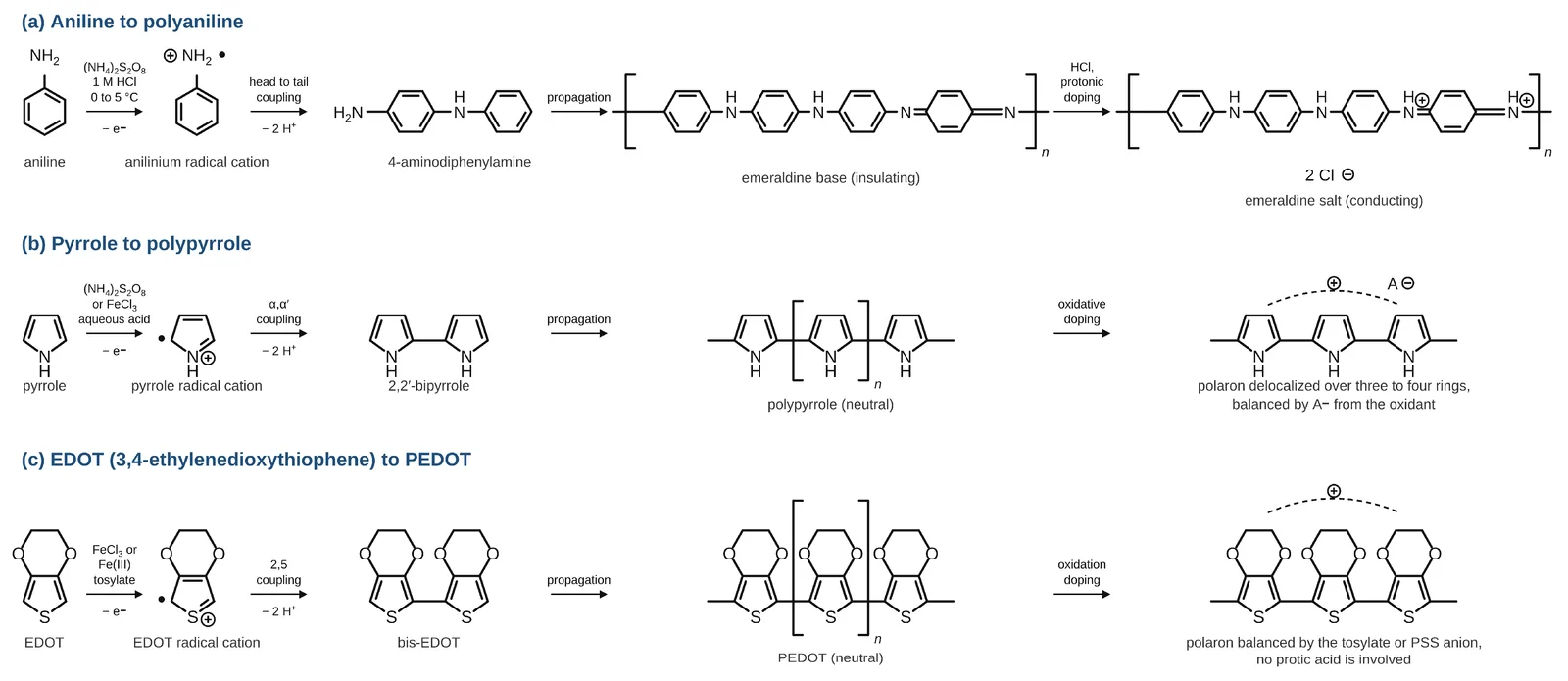
Chemical oxidative polymerization of conductive polymers. (**a**) Aniline is oxidized by ammonium peroxydisulfate in acid to the anilinium radical cation, which couples head to tail to 4-aminodiphenylamine; propagation gives emeraldine base, which is converted to the conducting emeraldine salt by protonation of the imine nitrogens. (**b**) Pyrrole is oxidized to its radical cation, couples at the 2 and 5 positions with loss of two protons, and the resulting chain is doped oxidatively, the positive charge being delocalized over three to four rings and balanced by an anion from the oxidant. (**c**) EDOT is oxidized by iron(III) and couples at the 2 and 5 positions to give PEDOT, which is doped by oxidation rather than by acid, with tosylate or polystyrene sulfonate as the counter-ion. Original figure prepared by the authors.

Figure 2 sets out the mechanism for the three monomers treated here. In each case, one-electron oxidation gives a radical cation, two radical cations couple with the loss of two protons, and repeated oxidation and coupling extends the chain. The routes differ in where the chain forms and in how the charge carriers are introduced. Aniline couples head to tail through nitrogen, and the product is doped by protonation of the imine nitrogens, which is reversible with pH. Pyrrole and EDOT couple at the 2 and 5 positions and are doped oxidatively, the charge being balanced by an anion supplied by the oxidant or the added dopant rather than by a protic acid.

### 2.2. Variants and Templating Approaches

Beyond conventional bulk mixing, several tailored polymerization techniques have been developed. In situ polymerization involves polymerizing the monomer directly on a substrate or template. For example, aniline can be polymerized in the presence of cellulose or cotton fibers, coating them with PANI. Joseph et al. demonstrated that vacuum-filtering aniline onto cotton or nylon fabric and initiating oxidative polymerization produces a dense PANI nanofiber coating on each fiber (seen in SEM) [25]. This in situ route yields conductive textiles with uniform coating and mechanical integrity. Interfacial polymerization is another powerful method: monomer and oxidant are separated into two immiscible phases (e.g., aniline in oil, APS in water). Polymer growth occurs at the oil–water interface, and the polymer precipitates as nanofibers on that interface. Konwar et al. used a vegetable-oil/water system to rapidly produce PANI nanofibers via interfacial oxidative polymerization [41]. In general, interfacial methods promote extended-chain conformations as chains grow at the interface [11]. A third approach is template-assisted growth, where physical scaffolds direct morphology. For instance, porous membranes or electrospun fibers can guide polymer deposition. Such templated polymerization can create well-ordered PANI nanotubes or nanofibers, effectively transferring the template geometry. However, templating often requires post-processing and gives lower yields than bulk methods [39]. Finally, chemical polymerization has been adapted to fiber spinning: one can dissolve doped polymer or a polymer salt complex and wet spin it into a coagulating bath. For example, Fang et al. developed a solvent-exchange wet spinning method for PANI: a CSA-doped PANI solution in *m*-cresol was extruded into DMF, slowly exchanging solvent and drawing ultrafine fibers. In that process, controlling solvent miscibility and draw ratio yielded continuous PANI fibers below 5 µm in diameter [33]. Thus, several chemical routes, namely in situ coating, interfacial reaction, templating and solution spinning, can be used to fabricate one-dimensional CP architectures.

**Figure 3:**
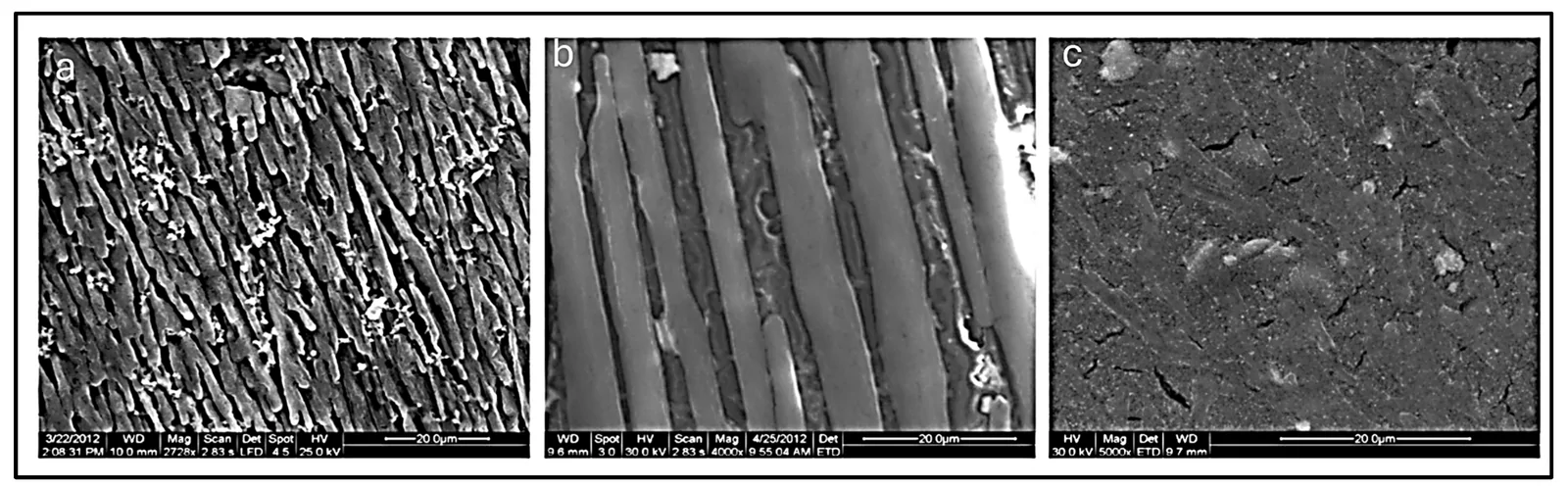
Scanning electron micrographs of conductive polymer nanofibers obtained by template-free chemical routes. (**a**) PANI–AuNP, (**b**) polythiophene–AuNP, (**c**) and PEDOT–AuNP composites. The PANI fibers are approximately 100 nm across, thinner than PT or PEDOT under comparable conditions. Reproduced from [42] under CC BY 4.0.

Template free chemical routes can also embed metal nanoparticles directly within the fibers as they form: Figure 3 compares PANI, polythiophene and PEDOT nanofibers decorated with gold nanoparticles, the PANI fibers being the finest at approximately 100 nm [42].

### 2.3. Morphology Control and Scale-Up

Fiber morphology in chemical polymerization is highly sensitive to reaction parameters. The monomer concentration is a key factor: diluted monomer solutions favor one-dimensional growth, whereas high concentrations promote bulk aggregates. Wang et al. systematically showed that low aniline concentration yields abundant PANI nanofibers, but higher concentration leads to thicker rods or granules [43]. Similarly, the acidity (pH) dictates chain structure: weak acid yields oligomeric, phenazine-containing PANI that stacks into sheets, while strong acid induces linear chains that entangle into nanofibers. In practice, increasing HCl concentration lengthens the conjugation and enhances doping, which also aids fiber formation. The oxidant ratio (aniline: APS) further tunes the nanofiber branching. Qiu et al. found that more APS (relative to aniline) increased nucleation sites, producing shorter, more-branched PANI nanofibers [39]. In all cases, rapid mixing or seeding tends to create more uniform fibers, whereas slow mixing gives irregular agglomerates. For instance, Rahman et al. recently reported that a rapid-mixing polymerization of PANI produced thin, uniform, interconnected nanofibers, whereas conventional stirring gave thicker, less uniform fibers [44].

These methods can be scaled: chemical polymerization is inherently high-yield and adaptable to large volumes [39]. Li et al. note that bulk oxidative polymerization readily produces kilogram-scale PANI nanofibers [40]. Indeed, Fang et al. spun more than 5 km of ultrafine PANI fiber in a two-hour run by optimizing solvent exchange and spinning conditions [33]. In contrast, template methods (e.g., using hard molds) generally limit scale. Overall, careful control of concentration, pH, temperature and mixing allows tailoring fiber diameter, length and porosity, and chemical polymerization can be scaled to industrially relevant batches by continuous stirring or flow reactors [33,43].

### 2.4. Attainable Properties

The chemical route accesses a wide property range, but that range is bounded in a characteristic way. When fully doped, PANI and PPy typically reach conductivities of 10^0^ to 10^2^ S/cm measured on dense, compacted material, the exact value depending on dopant identity, oxidation state and post-treatment; uniform doping and higher crystallinity consistently improve transport [39]. Microwave-assisted polymerization illustrates the sensitivity of the route: Qiu et al. showed that microwave-synthesized PANI reached 7 to 10 times higher electrical conductivity than PANI prepared by the conventional method under the same acid and oxidant conditions, attributed to narrower chain-length distributions and increased crystallinity [39]. PEDOT-based material reaches comparable or higher values because PSS acts as both dopant and template. Doping level can be adjusted after synthesis by acid treatment or reversed by base, although repeated cycling degrades mechanical integrity.

Mechanically, the products are stiff and brittle, and the reported values divide sharply according to how the fiber was formed rather than how the polymer was made. Material precipitated directly from a polymerization bath is reported as powders, mats and coatings, and tensile data are rarely given at all. For PPy nanotubes, AFM-cantilever measurements give elastic moduli of the order of GPa, indicating stiff but inherently brittle structures [37]; tensile strength and elongation at break for bulk PPy coatings are generally low, so most practical constructions rely on a textile or elastomeric support. The outlier is wet-spun PANI, which reaches approximately 1080 MPa tensile strength and 30 GPa modulus below 5 µm diameter [33]. That value is achieved by extrusion into a coagulation bath under a controlled draw ratio, so it demonstrates what drawing contributes rather than what polymerization contributes.

Electrochemically, the route performs well where accessible surface area is generated by the reaction itself. Interfacial polymerization at a vegetable-oil/water boundary yields nanofibers suitable for direct use as symmetric supercapacitor electrodes without templates or halogenated solvents [41], and altering only the mixing regime doubles areal capacitance to approximately 1000 mF/cm^2^ [44]. In situ growth on a pre-formed substrate extends the same chemistry to composites, giving lightweight conductive aerogel fibers for strain and temperature sensing [45] and coated textiles with stable 11 to 15 dB shielding across 8 to 18 GHz [25]. In each case, the conducting phase is the achievement; the supporting geometry comes from something else, whether a spinneret, a template or an existing fabric.

### 2.5. Sources of Batch Variability

The parameter sensitivities described in Section 2.3 are also the reason that chemical polymerization is difficult to repeat exactly. Four mechanisms account for most of the reported inconsistency. The first is oxidant depletion. Because both the oxidant and the monomer are consumed as the reaction proceeds, and because the reaction is strongly exothermic, material formed early in a batch experiences a different local environment of oxidant availability and temperature from material formed late. Since that ratio controls nucleation density and branching [39], a single batch can contain fibers grown under different conditions, which broadens the diameter distribution without any change in the nominal recipe.

The second is uncontrolled secondary nucleation. Fiber formation in aniline polymerization depends on nuclei forming rapidly and then growing rather than on new nuclei appearing throughout the reaction. Where mixing is slow or local concentration gradients persist, fresh nuclei continue to appear, and the product shifts from nanofibers toward rods and granules [43]. This is why rapid mixing and seeding give more uniform products than conventional stirring [44], and it also explains why the same protocol can give different morphologies in vessels of different geometry.

The third is thermal control. Oxidative polymerization of aniline and pyrrole is strongly exothermic, so the internal temperature of a batch depends on its volume and on the efficiency of cooling. Because acidity, chain length and doping level all respond to temperature, a protocol validated at small scale does not transfer directly to a larger vessel. The fourth applies specifically to in situ routes, where the substrate itself is a variable. Surface chemistry, fiber packing density and residual finish on a textile all affect how monomer adsorbs and where polymerization initiates, so nominally identical coatings on different fabric lots can differ in coverage and adhesion.

The practical consequence is that reproducing a chemically polymerized morphology requires matching monomer concentration, acidity, oxidant ratio, temperature history, mixing regime and substrate condition simultaneously. Few published procedures report all six. This is a different situation from electrospinning, where the corresponding variables are fewer, are instrument settings rather than reaction outcomes, and are conventionally reported.

## 3. Electrospinning

**Figure 4:**
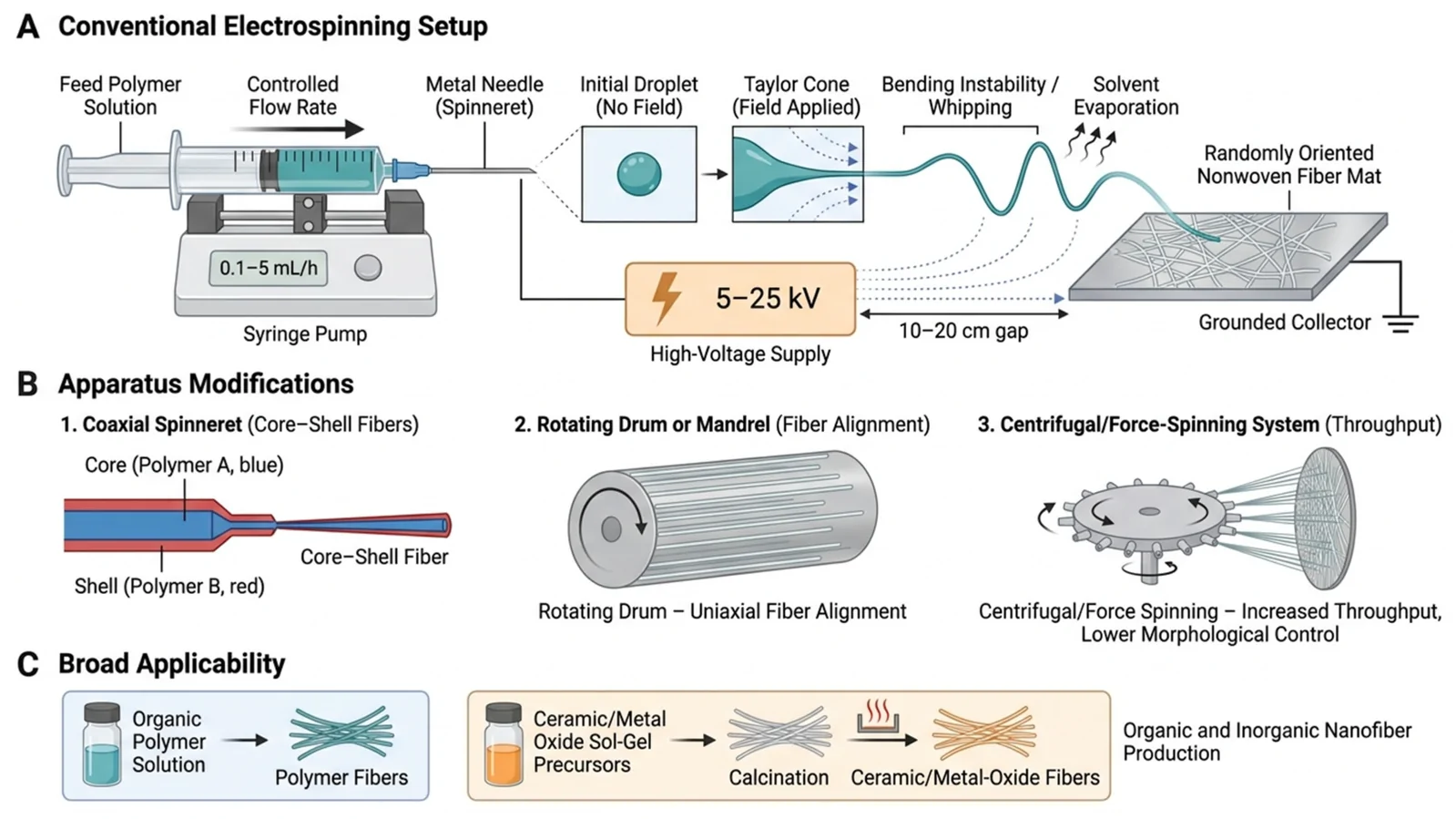
Electrospinning apparatus and its principal variants. (**A**) Conventional single needle setup showing the syringe and polymer solution, the high voltage supply and the collector; (**B**) configuration variants, including the coaxial needle used for core shell fibers, the rotating mandrel or drum used to align fibers, and centrifugal or force spinning systems used to raise throughput; (**C**) the breadth of feedstock the technique accepts, covering organic polymer solutions, which give polymer fibers directly, and ceramic or metal oxide sol–gel precursors, which give inorganic fibers after calcination.

Electrospinning fabricates polymer fibers by drawing a charged jet from a polymer solution in a strong electric field. A conventional apparatus comprises four elements (Figure 4A): a syringe holding the feed solution, a syringe pump delivering it at a controlled flow rate, a metal needle or spinneret, and a grounded or oppositely charged collector, with a high-voltage supply operating typically between 5 and 25 kV across a needle-to-collector gap of 10 to 20 cm [46,47,48,49]. Without an applied field, the solution simply forms droplets at the needle tip under surface tension [50]. Once the field is applied, charge accumulates in the solution and electrostatic repulsion distorts the droplet into a Taylor cone; above a threshold voltage, a jet is ejected from the cone apex, undergoes bending instability and rapid whipping, and thins by orders of magnitude while the solvent evaporates [51]. Dry solid fibers deposit on the collector as a randomly oriented nonwoven mat whose thickness increases with spinning time [28]. The basic configuration is readily modified (Figure 4B): coaxial spinnerets produce core–shell fibers, rotating drums or mandrels impose uniaxial alignment, and related centrifugal or force-spinning systems raise throughput at the cost of morphological control [47,52,53,54]. Beyond organic polymers, the same arrangement has been used to produce ceramic and metal-oxide nanofibers by spinning sol–gel precursors and calcining the resulting mats [28,55,56,57].

This breadth extends beyond organic polymers. The same apparatus spins ceramic and metal oxide fibers from sol–gel precursors, which are converted by subsequent calcination (Figure 4C), so the geometric control described below is not restricted to a single material class [28,55,56,57].

### 3.1. Strategies to Make Conductive Fibers

Conventional conjugated polymers (CPs) often cannot be electrospun on their own due to low molecular weight and rigidity, so various strategies have been developed to create conductive fiber mats. Blend electrospinning is the simplest approach: the CP (or a dopant) is dissolved or dispersed in a spinnable polymer solution. For example, doped polyaniline (PANI) can be blended with carrier polymers such as poly(ethylene oxide) (PEO) or polystyrene (PS) to allow fiber formation [4]. In reported cases, PANI/PEO blends yielded fibers 0.9 to 1.9 µm in diameter [58], while PANI/PS blends produced much thinner fibers (72 to 100 nm) [59]; in both cases, fiber conductivity increased with PANI content (though it remained slightly below that of equivalent cast films). Likewise, adding trace amounts of very-high-MW PEO (0.5  wt%) [60] enabled electrospinning of PEDOT:PSS into smooth 300 to 400 nm fibers, yielding single-fiber conductivity as high as approximately 35 S/cm [4].

Another approach is coaxial (core–shell) electrospinning. This uses a concentric dual-needle [53] spinneret to form fibers with a core and an outer shell [4,28,54]. In one style, the core can be a CP dissolved in a volatile solvent, while the shell is a high-MW polymer that can be removed after spinning. For example, emeraldine-base PANI in chloroform was used as a core with PMMA in DMF as a sacrificial shell; after electrospinning and solvent removal of PMMA, the remaining PANI fibers were much thinner approximately 620 nm and highly conductive [4]. Coaxial spinning thus enables “neat” CP fibers by providing the necessary viscoelastic support during spinning, and a variety of core shell combinations have been demonstrated.

### 3.2. Morphology, Alignment and Tuning

The structure and architecture of electrospun mats can be tuned by adjusting spinning parameters and collector design. For example, increasing polymer concentration (and thus solution viscosity) generally produces thicker fibers: low concentration gives beads or no fibers, whereas an optimal concentration yields bead-free nanofibers [61]. Systematic studies show that fiber diameter can be smoothly varied by changing solution properties and field strength. Meanwhile, the intrinsic porosity of electrospun mats (due to the random network of fibers) gives a high surface area [61]. Individual fiber porosity can also be tailored through solvent choice and ambient humidity, which can induce pores on the fiber surface.

Fiber alignment and overall mat architecture depend mainly on the collector. A stationary flat collector produces a random, nonwoven network resembling the interpenetrating 3D collagen matrix of the extracellular matrix [28,62]. In contrast, using a rotating mandrel or drum at high speed induces uniaxial alignment: above a critical rotation velocity (typically several m/s), fibers become highly aligned in the circumferential direction [61,63]. Alignment can also be achieved using parallel electrodes (gap collectors) or auxiliary electrodes, where fibers span across a gap or follow electric field lines [64]. Larger electrode gaps yield better alignment up to a limit (beyond which very thin fibers may break under their own weight) [65]. Aligned mats and patterned collectors allow engineering of anisotropic architectures (e.g., gradient or wavy alignments) for advanced devices. By controlling these factors, one can produce mats ranging from random nonwoven meshes to highly ordered, 3D nanofiber scaffolds.

### 3.3. Post-Treatments and Functionalization

After electrospinning, conductive fiber mats are often modified to improve morphology and charge transport. Thermal annealing (e.g., 100 to 200 °C) can crystallize polymer chains and remove residual solvent, enhancing fiber uniformity. Post-treatment with polar solvents or acids is common, and two chemically distinct effects are involved. Secondary doping with a high-boiling polar solvent such as ethylene glycol or dimethyl sulfoxide does not alter the oxidation level of the polymer; it removes excess insulating PSS and improves the ordering of the PEDOT chains, raising carrier mobility and interdomain connectivity rather than carrier concentration [13,66]. Exposing as spun PEDOT:PSS fibers to dimethyl sulfoxide in the vapor phase, rather than immersing them in the liquid, allows the solvent to permeate the fiber without dissolving it or fusing adjacent fibers at their crossing points, and it has been reported to raise conductivity by approximately two orders of magnitude [35]. Protonic doping is the second effect and applies to PANI and PPy, in which a strong acid protonates the backbone to generate polarons and raises carrier concentration directly. Hot sulfuric acid treatment of PAN/PEDOT:PSS mats also raises conductivity, but there the acid acts by stripping excess PSS and promoting crystallization rather than by protonating the backbone [67]. Vapor-phase polymerization (VPP) is another approach: a template fiber (e.g., PLA or PCL) loaded with oxidant is exposed to EDOT vapor, forming a conformal PEDOT shell. Pires et al. reported aligned PLA/PEDOT fibers made by VPP that achieved 1.5 × 10^−1^ S/cm [68]. Finally, surface coatings with conductive inks, metal nanoparticles or carbon (e.g., CNT, graphene oxide) can be applied to fibers. In each case, these post-treatments generally reduce fiber diameter variation and inter-fiber junction resistance, yielding increases in electrical conductivity and improved device function [35,68].

### 3.4. Attainable Properties and Representative Examples

Recent studies illustrate diverse polymer systems and methods. Polymer blends are common: for instance, PAN/PEDOT:PSS mats (Clevios^TM^ PH1000, Heraeus, Leverkusen, Germany) that were electrospun and then heat-annealed (240 °C) followed by hot H_2_SO_4_ treatment yielded fiber diameters from 440 to 940 nm and conductivity of 3.2 × 10^−3^ S/cm. This exceeds as-spun PEO:PEDOT:PSS mats (1.2 × 10^−4^ S/cm at mat level) and as-spun PVA:PEDOT:PSS mats (9 × 10^−6^ S/cm at mat level) [67]. All three values are mat-level four-point measurements and are therefore not directly comparable with single-fiber data from the same materials. Coaxial or side-by-side architectures can also boost mat-level performance: Liu et al. produced PEO/CSA-doped PANI fibers with a Janus structure; side-by-side mats had conductivities 8 to 15× higher (10^−5^ to 10^−4^ S/cm) than equivalent blended PANI/PEO mats [69], both measured across the full mat. Wet-spun PVA/PEDOT:PSS filaments reached above 10 S/cm after methanol coagulation, a different geometry and processing state that accounts for the much higher value [66,70]. In summary, these examples show that conductivity depends on polymer choice, processing and, critically, on measurement geometry. Mat-level values for as-spun blends fall between 10^−6^ and 10^−2^ S/cm, while single-fiber or post-converted values reach 10^0^ to 10^1^ S/cm for the same class of material (Section 4.1). Reporting a single conductivity range without specifying geometry conflates these distinct quantities.

Neat PANI is difficult to electrospun on its own, so a carrier such as poly(ethylene oxide) or silk fibroin is normally blended into the dope to obtain a spinnable solution [71,72,73]. Simotwo et al. electrospun 93% PANI with 7% PEO into free-standing nanofiber mats that reached specific capacitances of approximately 308 to 385 F/g with good cycle retention [73], which illustrates that the porous three-dimensional network produced by electrospinning facilitates ion and electron transport [73,74].

### 3.5. Post-Spinning Conversion to Conducting Fibers

**Figure 5:**
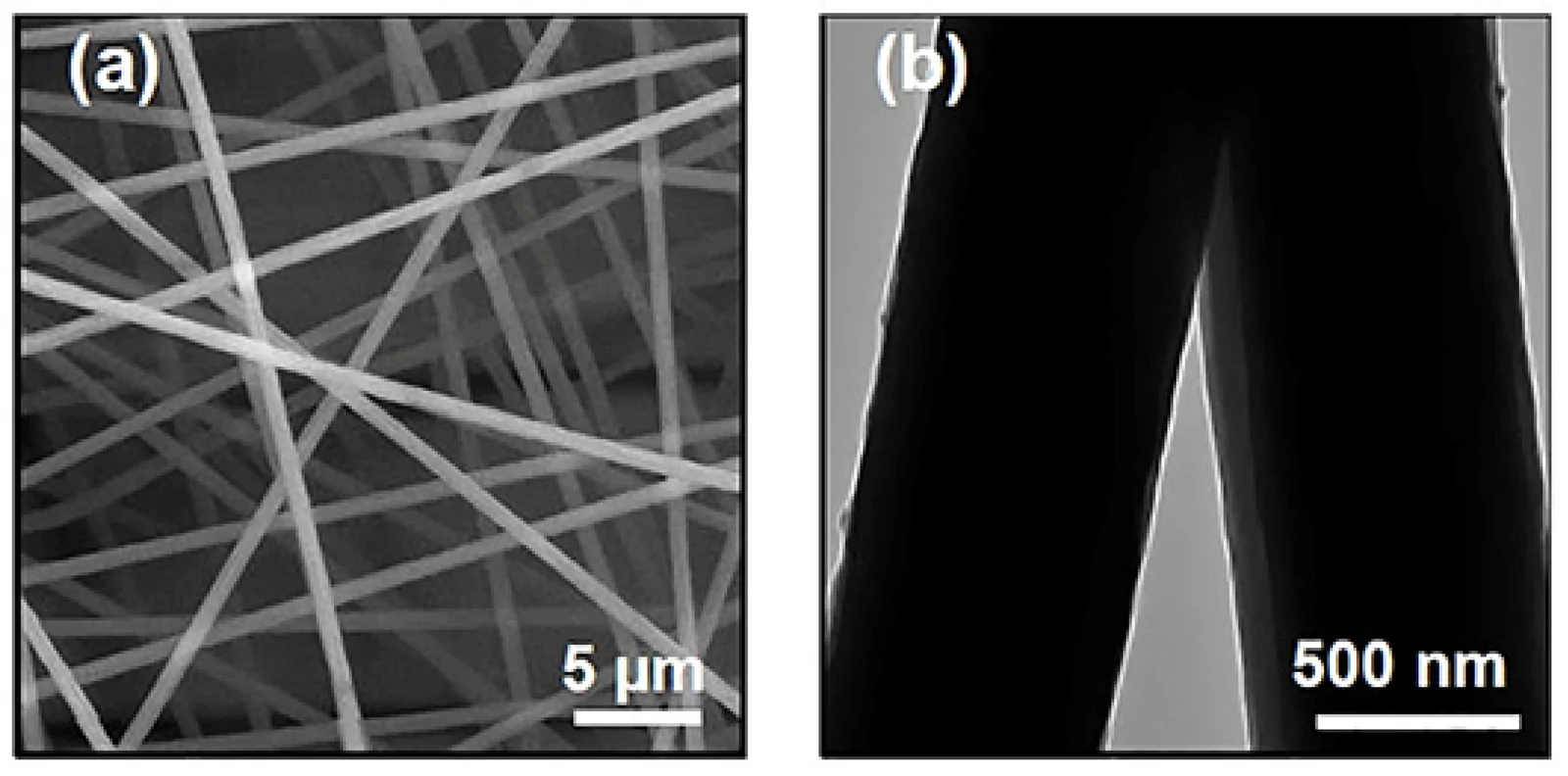
SEM (**a**) and TEM (**b**) images of electrospun polyacrylonitrile (PAN) nanofibers used as templates for subsequent conducting-polymer growth. Reproduced from [36] under CC BY 4.0.

The routes above place the conductive polymer in the spinning solution, which caps conductivity at the loading the solution will tolerate. The alternative is to spin an inert or sacrificial template first and form the conductive polymer afterwards, so that the architecture is set by electrospinning and the conducting phase by chemistry. Sapountzi et al. electrospun polyacrylonitrile (PAN) nanofibers (Figure 5) and grew polypyrrole on them by vapor-phase polymerization to give PAN/PPy core–shell fibers for glucose biosensing [36]. Electrospun polyurethane or cellulose mats have similarly been dipped in monomer/oxidant solutions so that PANI or PPy grow in situ on the fibers, and PEDOT has been polymerized onto electrospun nitrile-butadiene rubber/PEGDMA fibers to reach approximately 6 S/cm while the mat remained stretchable [4]. Where the template is removed afterwards, as when PVP fibers loaded with ferric toluate are exposed to EDOT vapor and then rinsed, hollow PEDOT nanofibers of approximately 350 nm remain and the mats reach 60 S/cm [4], within the range of chemically derived bulk material.

Three practical considerations govern whether this works. The first is oxidant management: in vapor-phase polymerization, the oxidant is spun into the template fiber, so its loading sets both the thickness and the doping level of the resulting shell, and excess residues must be rinsed out or they act as ionic impurities that degrade long-term stability [35,36]. The second is adhesion between the conducting shell and the template; where the two are chemically dissimilar, the shell delaminates under bending and the mat loses conductivity long before it loses mechanical integrity, which is why nitrile-functional and surface-oxidized templates are preferred over inert polyolefins [4,35]. The third is whether the template is retained or sacrificed. Retaining it preserves the mechanical properties of the mat and keeps processing short, but the insulating core limits volumetric conductivity; removing it gives higher intrinsic conductivity at the cost of an extra rinsing step and a more fragile product [4].

These routes are strongest where accessible surface area matters more than bulk conductivity, namely biosensing electrodes, neural interfaces and fiber-shaped supercapacitors, and weakest where a continuous mechanically robust conductor is needed, such as interconnects in e-textiles [16,35,36]. They also expose a gap in the literature: very few studies report the conductivity of a converted mat before and after mechanical cycling or washing. Those that do, such as field testing of a narrow fabric e-textile system [75] and a dry-wash durability study on conductive textiles [76], represent isolated examples rather than a systematic body of evidence, so the durability advantage often claimed for hybrid fibers over simple coatings remains largely unverified.

### 3.6. Sources of Process Variability

Electrospinning is not free of variability, and stating its sources precisely is necessary before the reproducibility comparison in Section 5. The dominant environmental variable is relative humidity, which governs both charge dissipation from the jet and the rate of solvent evaporation, and therefore influences almost every property of the collected fiber [77]. High humidity slows evaporation, extends the flight time over which the jet can thin, and can trigger vapor induced phase separation that leaves pores on the fiber surface. Low humidity accelerates skin formation and can produce wrinkled or buckled morphologies. Because ambient laboratory humidity varies seasonally and between rooms, an uncontrolled setup can produce different fiber populations from the same solution on different days, and stable atmospheric conditions are accordingly regarded as a prerequisite for repeatable results [77].

Three process variables account for most of the remaining scatter. Jet instability is intrinsic to the technique, since the whipping motion that thins the fiber is a chaotic bending instability rather than a steady flow, so fiber diameter is a distribution rather than a single value even under fixed conditions. Needle clogging arises when solvent evaporates at the spinneret tip and deposits solid polymer, which progressively alters the effective orifice diameter and the flow field during long runs. Solution aging matters for conductive polymer systems in particular, because doped PANI and PEDOT:PSS dispersions change in viscosity and aggregation state over hours, so the spinnability of a solution is itself time-dependent.

The distinguishing feature of these sources is that each is an instrument or environment variable that can be measured and held constant. Humidity can be controlled in an enclosed chamber, clogging can be suppressed by coaxial sheath flow or periodic tip cleaning, and solution age can be recorded. This is what separates the two routes in practice. Electrospinning variability originates in conditions external to the material and is therefore addressable by instrumentation, whereas chemical polymerization variability originates in the reaction itself and is addressable only by controlling the chemistry more tightly.

## 4. Characterization Techniques for Fibers

To compare polymerization routes, microstructural imaging is essential. Scanning electron microscopy (SEM) is a fundamental tool for fibrous mats, yielding detailed topography and fiber dimensions [78]. In electrospun mats, SEM readily reveals uniform fiber diameters, alignment and interstitial voids [79]. For example, SEM micrographs of uncoated polyester fibers vs. PEDOT-coated polyester threads show markedly increased surface roughness relative to the uncoated substrate [80]. At higher resolution, transmission electron microscopy (TEM) provides internal structural detail, such as core–shell morphologies or nanoparticle inclusions [81]. Atomic force microscopy (AFM) similarly maps nanoscale surface roughness and mechanics on single fibers. X-ray diffraction (XRD) is used to probe crystallinity: electrospinning often yields broad, low-intensity peaks due to rapid solidification, while chemical polymerization can leave more ordered domains. In practice, XRD has shown that many electrospun hybrid fibers become largely amorphous dispersions during fabrication [79].

Surface area and porosity are quantified by adsorption and porosimetry methods. The Brunauer Emmett Teller (BET) gas adsorption technique is routinely used to determine specific surface area from nitrogen isotherms [82]. For a smooth solid cylinder, the external specific surface area follows directly from geometry as four divided by the product of the polymer density and the fiber diameter, which gives approximately 33 m^2^/g for a 100 nm fiber of density of 1.2 g/cm^3^. Values substantially above this require internal porosity or surface roughness rather than a small diameter alone, and measured values for conductive polymer fibers are consistent with the geometric estimate: PANI-coated PAN nanofibers gave a BET area of approximately 16.8 m^2^/g against 9.1 m^2^/g for bare PAN fibers [83]. Four quantities are reported interchangeably in this literature and should be distinguished. The external surface area of the fibers is set by diameter and density. The internal porosity of the fiber wall is set by the processing route. The inter-fiber void fraction of the mat, which approaches 90% in electrospun mats [84], governs electrolyte access and is not measured by BET at all. The BET area is the sum of the external and accessible internal surfaces as probed by the adsorbate and is the only one of the four that is measured directly. Likewise, adjusting processing (e.g., sonication or doping) can yield nanofiber mats with varied BET areas [85,86]. SEM images complement BET by providing pore-size estimates [78]. Larger pores and interconnected channels can be measured by porosimetry (mercury intrusion or capillary flow) according to ASTM standards. Together, these techniques provide a thorough picture of fiber morphology and porosity in both chemically polymerized and electrospun conductive polymer fibers [79,82,83].

### 4.1. Measurement Geometry and the Comparability of Reported Conductivities

**Figure 6:**
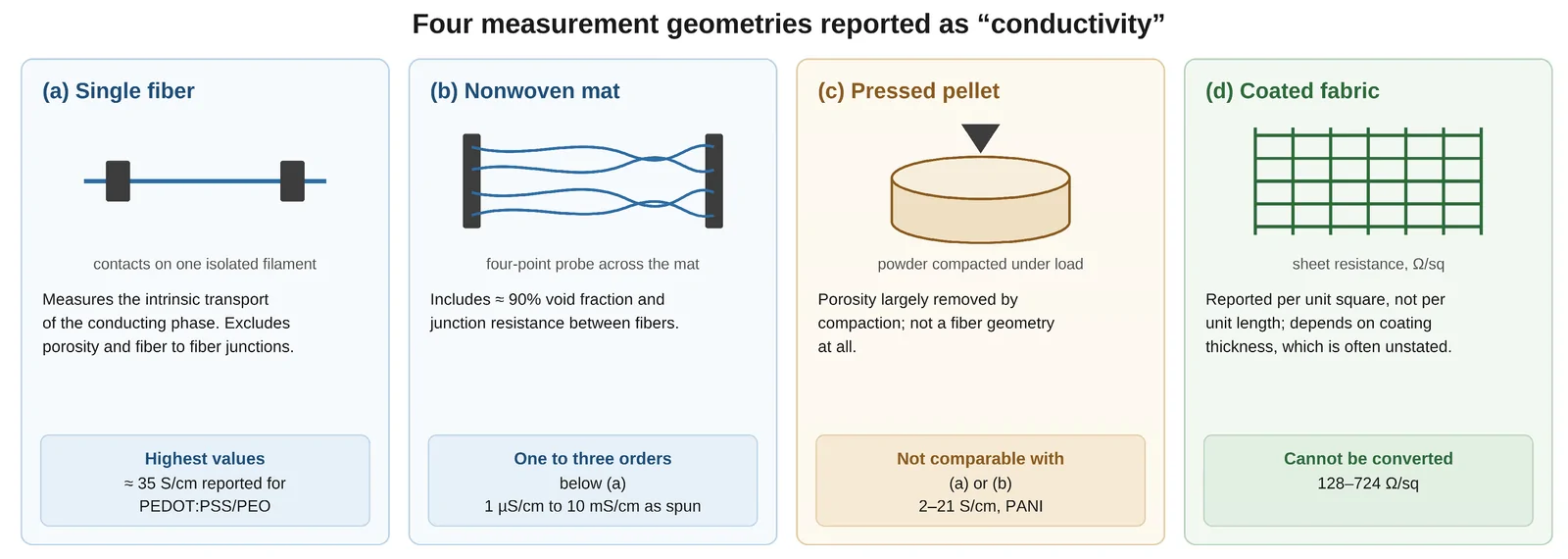
Measurement geometries used to report conductivity of conductive polymer fibers. (**a**) Single filament with direct contacts, with a representative value of approximately 35 S/cm for an electrospun PEDOT:PSS/PEO fiber [4,60]; (**b**) four point probe across a nonwoven mat, whereas spun blends fall between 1 µS/cm and 10 mS/cm; (**c**) pressed pellet of bulk polymer, 2 to 21 S/cm for chemically polymerized PANI, Perdigão et al., 2020; (**d**) coated fabric characterized as sheet resistance, 128 to 724 Ω/sq, He et al., 2013. Values obtained in different geometries are not interchangeable, and the same material can differ by one to three orders of magnitude between (**a**) and (**b**). Original figure prepared by the authors.

Before any electrical comparison can be made, a problem in the literature has to be stated plainly. Conductivity is reported for CP fibers in at least four incompatible geometries, illustrated in Figure 6, and values from them are routinely quoted side by side as though interchangeable. Single-fiber measurements place contacts on one isolated filament and therefore report the intrinsic transport of the conducting phase, excluding porosity and fiber to fiber junction resistance (Figure 6a). Mat-level four-point measurements include both, and since electrospun mats have void fractions near 90% [84], a mat is expected to read well below the single-fiber value for the same material (Figure 6b). The one- to three-order-of-magnitude figure quoted in this review is the span between the single-fiber and mat values found across the surveyed literature, not a ratio measured on one material in both geometries; no such paired measurement was identified. A single-fiber value is also not the intrinsic conductivity of the conducting phase unless the fiber is neat. For a composite or coated fiber, it is an effective value for the whole cross-section, and the area used to normalize it, whether the full fiber cross-section or that of the conducting shell alone, changes the result by the ratio of those areas and is frequently unstated. The normalization used is recorded wherever the source gives it. Pressed pellets remove porosity by compaction and are not a fiber geometry at all (Figure 6c). Coated fabrics are reported as sheet resistance in ohms per square (Ω/sq), which cannot be converted to S/cm without a coating thickness that is frequently unstated (Figure 6d).

The consequences for a method comparison are direct. An electrospun PEDOT:PSS blend fiber measured individually reaches approximately 35 S/cm [4,60], while as-spun mats of comparable blends are reported between 10^−6^ and 10^−2^ S/cm; both figures are correct and they describe the same class of material. Much of the apparent electrical deficit of electrospinning relative to chemical polymerization is therefore a consequence of comparing mats against pellets. Conductivities in this review are accordingly compared as orders of magnitude within a stated geometry, and the geometry is given explicitly wherever a value is quoted, with the measurement conditions stated. A recent study illustrates the standard that this implies in reporting conductivity for coaxially electrospun PAN/PANI mats containing graphene oxide nanoscrolls, Bashir and Depan specified the strip dimensions, the inner probe separation, four-wire operation and the resistivity relation applied, and they verified ohmic contact from the linearity of the current voltage response [87]. Reporting conductivity without naming the measurement configuration should be regarded as an incomplete result.

### 4.2. Electrical and Electrochemical Characterization

Electrical characterization of conductive polymer fiber mats uses the same fundamental techniques as for bulk films, but the choice of geometry matters for the reasons set out in Section 4.1. For electrospun mats, the standard approach is a four-point probe placed directly on the mat surface; this measures mat-level sheet conductance, which includes fiber-to-fiber junction resistance and the mat’s high void fraction. Where single-fiber values are sought, a microprobe or conductive-AFM configuration isolates one filament. The difference between the two configurations is one to three orders of magnitude for the same material, so both the method and the contact spacing must be reported. Electrochemical impedance spectroscopy (EIS) quantifies charge-transfer resistance and capacitance, and cyclic voltammetry (CV) identifies the doping and dedoping transitions of PANI and confirms that the redox-active phase is intact after processing. For chemically polymerized coatings on textiles, a four-point probe yields sheet resistance in Ω/sq, which requires a stated coating thickness to convert to S/cm; because thickness is frequently not reported, the two quantities are kept separate throughout this review [88].

### 4.3. Mechanical and Durability Testing

Mechanical characterization of conductive polymer fiber mats is performed at two scales. At the mat level, a universal testing machine (e.g., Instron 3365, Instron, Norwood, MA, USA) pulls a rectangular coupon of the nonwoven mat to failure, yielding tensile strength, elongation at break and elastic modulus; an extensometer or digital image correlation tracks strain. For electrospun mats, this test also captures how the CP coating changes the mechanical response relative to the bare carrier: in practice, PANI or PPy coatings stiffen the mat but can reduce elongation at break by embrittling the fibers. At the single-fiber level, micro-tensile rigs and AFM-based nanoindentation give modulus and hardness, though such measurements are technically demanding and appear in a minority of published studies. Bending or cyclic-flexure rigs flex the mat repeatedly while monitoring electrical resistance, revealing how many deformation cycles can be sustained before conductivity loss exceeds a defined threshold. For wearable applications, standardized laundry testing to ISO 6330 [89], which specifies aqueous domestic washing at 40 to 60 °C, is a further requirement. Results obtained by simulated dry cleaning are not equivalent to it: a conductive textile sustained 40 simulated dry-wash cycles with no measurable conductivity loss [76], but dry cleaning is a solvent process that does not expose the dopant to water, detergent and mechanical agitation in the way that aqueous laundering does. The persistent weakness across the literature is that very few studies combine all four measurements, namely mat tensile, single-fiber, bending endurance and wash cycling, on the same specimen set [75,76].

## 5. Comparative Assessment: Materials and Methods

This section compares the two routes based on the three criteria set out in Section 1. Section 5.1, Section 5.2 and Section 5.3 first establish what is attributable to the choice of polymer rather than to the choice of route, since PANI, PPy and PEDOT:PSS differ in morphology, doping stability and compliance before any fabrication method is applied. Section 5.4, Section 5.5, Section 5.6 and Section 5.7 then compare the routes directly: qualitatively for PANI, then on reproducibility of geometry, on electrical performance with measurement geometry held constant, and on scalability.

### 5.1. Morphology and Structural Control

Conductive polymers exhibit diverse nanostructures depending on synthesis methods. For example, chemically polymerized polypyrrole (PPy) can form nanoparticles, nanotubes, or nanowires with controlled templates; ordered PPy nanotubes in particular offer large surface area and excellent mechanical strength [90]. Polyaniline (PANI) often grows as granular or fibrous aggregates, and introducing surfactants or dopants such as camphorsulfonic acid can tune PANI morphology into smooth nanofibers for uniform films [90,91]. Similarly, poly(3,4-ethylenedioxythiophene): PSS (PEDOT:PSS) typically forms globular domains that can crystallize into lamellar stacks when processed with high-boiling co-solvents, dramatically altering film morphology and ordering. In situ interfacial templating has likewise produced flexible PPy membranes with aligned nanotubes, yielding a robust, porous structure [90]. Such structural control enables tailoring of surface area, charge carrier pathways, and composite interfacial contact, which are critical for device performance [90,92].

### 5.2. Electrical Performance and Doping Stability

The various polymers differ markedly in electrical conductivity and dopant behavior. PEDOT:PSS exhibits especially high baseline conductivity (often around 1 to 10 S/cm) and can be boosted by orders of magnitude by adding polar solvents (e.g., DMSO or EG) during film formation [91]. In contrast, undoped PANI or PPy are initially insulating and require acid or anionic doping. For example, introducing anionic dopants into PPy nanoparticles raised their conductivity from only a few S/cm to over 20 S/cm [90]. PANI doped with strong acids (Cl^−^) also achieves much higher conductivity than with weaker dopants, so one study found chloride-doped PANI films had lower charge-transfer resistance than nitrate- or sulfate-doped analogues [93]. Overall, PEDOT:PSS-based devices tend to outperform PANI in conductivity and long-term operational stability [91,93]. Likewise, PPy coatings are often more stable against dedoping than PANI under ambient conditions [93,94]. Hybrid composites further enhance performance: for instance, a PEDOT–PPy thin-film electrode showed superior electrochemical response and retained high conductivity and sensing reliability over extended cycling [95]. Nonetheless, even high-performance films degrade gradually under extreme strain or humidity, and conductive elastomers with intrinsically stretchable backbones are being explored to improve retention of conductivity under deformation [91,92].

### 5.3. Mechanical Properties and Flexibility

Three classes of mechanical data are reported for conductive polymers and should not be compared directly. The first is the intrinsic response of the pure polymer, which for PANI and PPy is stiff and brittle. The second is that of a thin conducting layer on a compliant substrate, where the measured compliance is largely that of the substrate: PPy-coated polyurethane films stretch to 300% elongation because the polyurethane does, and the modulus of the coated film is lower than that of the pure polyurethane because the coating is thin and discontinuous under strain [92]. The third is that of a composite in which the conductive polymer is a minority phase in an elastomer. Statements about flexibility in this review refer to the second and third classes unless the pure polymer is named. Within those classes the compliance is real and useful: conjugated chains are semi-flexible and accommodate bending without losing continuity [30], PEDOT:PSS films survive hundreds of bending cycles while retaining most of their conductivity, and composites of CPs with elastomers or ionic liquids achieve tensile strains over 100% while maintaining electrical function [30,92].

### 5.4. Chemical Polymerization Versus Electrospinning: PANI as a Case Study

Polyaniline (PANI) is a prototypical conductive polymer known for its ease of chemical synthesis, low cost, and high yield. Conventional chemical oxidative polymerization of aniline (e.g., using ammonium persulfate in acid) is straightforward and scalable, producing bulk PANI powders or films [96]. In contrast, electrospinning is a high-voltage fiber fabrication technique that produces continuous nanofiber mats. Electrospinning PANI typically requires dissolving or blending it with a carrier polymer (e.g., PVP) and a dopant (e.g., CSA) because PANI alone has poor solubility [96,97]. This yields fiber mats or yarns rather than bulk powder. Table 1 sets the two routes side by side across the aspects that matter most in practice.

### 5.5. Reproducibility of Fiber Geometry

**Figure 7:**
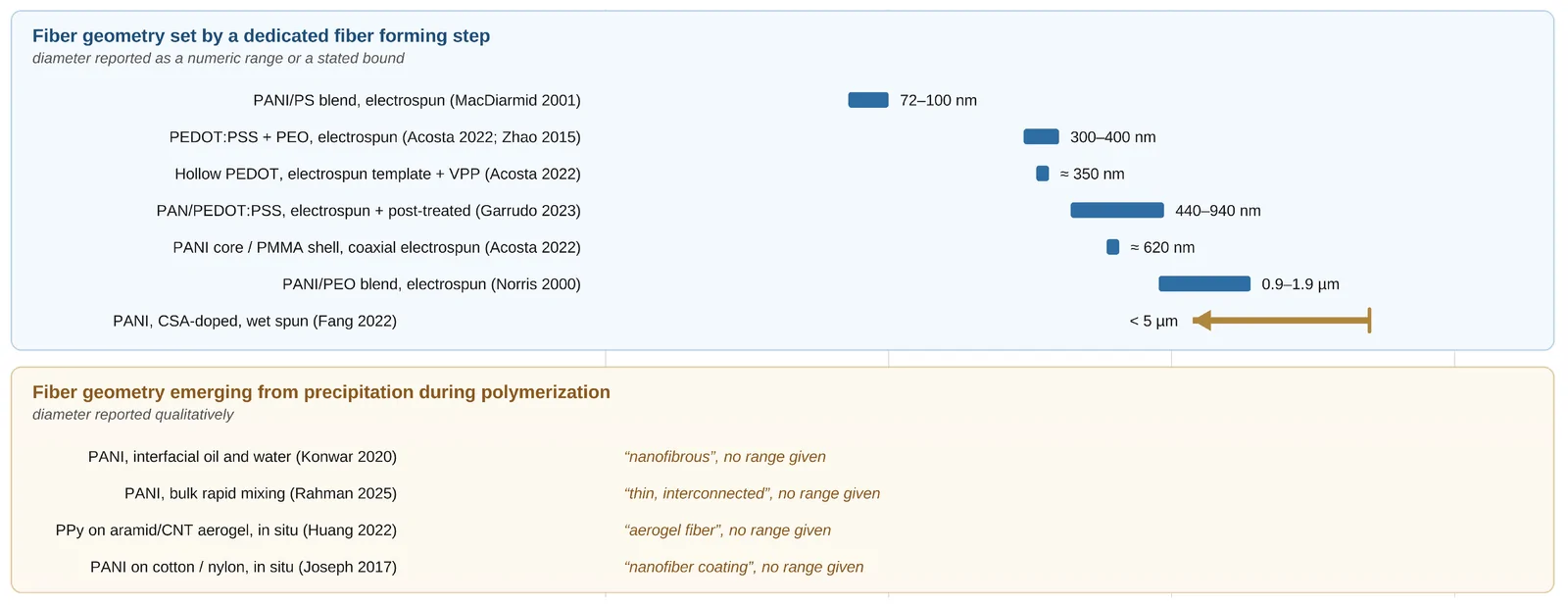
Fiber diameter as reported in the surveyed literature, grouped by fabrication route. Electrospun systems report numeric ranges set by process parameters; chemically polymerized systems report qualitative descriptions, with the exception of wet spinning, in which the fiber is formed by extrusion and drawing. Original figure prepared by the authors from the data compiled in Table 2.

Table 2 separates two things that this literature usually reports together: the chemistry by which the conductive polymer is formed and the operation by which the fiber geometry is established. The two are independent. Wet spinning is a fiber forming operation applied to a chemically polymerized polymer, and it is classified as such here rather than as a polymerization route. On that basis, the clearest difference between the routes is not a performance figure but a reporting pattern. Table 2 compiles fiber diameter, conductivity, measurement geometry and device performance for representative systems from each route, and Figure 7 plots the diameter column. Every electrospun system in the set specifies a numeric range: 72 to 100 nm for PANI/PS [59], 300 to 400 nm for PEDOT:PSS with a high-molecular-weight PEO carrier [4,60], approximately 350 nm for hollow PEDOT obtained by template conversion [4], 440 to 940 nm for post-treated PAN/PEDOT:PSS [67], approximately 620 nm for coaxially spun PANI after shell removal [4], and 0.9 to 1.9 µm for PANI/PEO [58]. Each of these was set deliberately through solution concentration, applied voltage, flow rate and collector configuration, and each can be targeted again from the stated parameters.

The chemically polymerized systems in the same table are described as nanofibrous [41], thin and interconnected [44], an aerogel fiber [45] or a nanofiber coating [25]. These are adjectives, not specifications. The distinction matters because it determines whether a result can be reproduced: a reader can attempt an electrospun geometry from the reported parameters, whereas reproducing a precipitated morphology requires matching monomer concentration, acidity, oxidant ratio, temperature and mixing history simultaneously, and small deviations shift the product from nanofibers to rods or granules [43]. Wang et al. showed that aniline concentration alone moves the product across that boundary [43], and Qiu et al. that the oxidant-to-monomer ratio controls branching [39]; these are useful levers, but ones that act on nucleation rather than on dimension directly.

Three distinct quantities are conflated in this literature and are separated here. Dimensional uniformity is the spread of fiber diameters within a single specimen, which a reported range describes. Intra-laboratory reproducibility is the agreement between specimens prepared from the same protocol in the same laboratory, which requires replicate batches to be reported. Inter-laboratory reproducibility is an agreement between laboratories executing the same protocol, which requires a round robin study. This survey found data for the first time only. No source compiled in Table 2 reports replicate batches, and no inter-laboratory comparison of conductive polymer fibers was identified. The observation of this review is therefore narrower than a claim about reproducibility: a diameter traceable to stated process parameters is reported wherever a dedicated spinning step defines the fiber, and a reader can attempt that geometry from the given parameters, whereas a morphology that emerges from precipitation during polymerization is generally described qualitatively and cannot be attempted in the same way. Whether either route transfers between laboratories are unknown. Two qualifications follow. First, chemical polymerization can and does produce fibers of controlled dimension: the template free route shown in Figure 3 gives PANI fibers of approximately 100 nm [42], and wet spinning gives fibers below 5 µm [33]. The distinction drawn here concerns what is reported, not what is attainable. Second, three of the electrospun entries in Table 2 are themselves described only as submicron or as aligned fibers, so the reporting pattern is a tendency within the surveyed set and not a rule.

A second and more consequential observation is that neither route has been subjected to an inter-laboratory comparison. No round robin study was identified in this survey in which the same protocol for a conductive polymer fiber was executed in multiple laboratories and the resulting diameters or conductivities compared. All reproducibility claims in the field are therefore within laboratory claims, and the more demanding question of whether a published protocol transfers between groups remains unanswered for both routes. This absence is itself a finding, and it is the strongest argument for the standardized reporting proposed in Section 7.

The single chemical entry that does specify a controlled dimension, wet-spun PANI below 5 µm [33], is also the only one in which the fiber is formed by a spinning operation. This is the central observation of the comparison. Reproducible fiber geometry tracks the presence of a dedicated fiber-forming step, not the choice of polymerization chemistry, and electrospinning is the most widely applicable such step for conductive polymer systems.

### 5.6. Electrical Performance at Constant Measurement Geometry

Once geometry is held constant, as set out in Section 4.1, the electrical picture changes. The frequently repeated claim that electrospinning yields poorer conductors conflates two separate effects: dilution by the carrier polymer, which is real, and the mat measurement geometry, which is not a property of the fiber. Three paired comparisons were identified in the surveyed literature, each varying one factor within a single study while holding composition and measurement geometry constant, and the argument rests on them. Thermal annealing followed by hot sulfuric acid raises PAN/PEDOT:PSS mats to 3.2 × 10^−3^ S/cm from the 10^−6^ to 10^−4^ S/cm of the untreated PEO- and PVA-based analogues in the same study [67]. A side-by-side Janus architecture improves on the equivalent blend by 8 to 15 times [69]. Rapid mixing doubles the areal capacitance of chemically polymerized PANI relative to conventional stirring [44]. Beyond these, the recovery of two to four orders of magnitude on carrier removal or post-spinning conversion is the span of values reported across different studies rather than a measured ratio: coaxially spun PANI after sacrificial shell removal, hollow PEDOT at 60 S/cm and PEDOT-converted rubber fibers at approximately 6 S/cm all fall within the range reported for chemically derived material [4]. The practical conclusion is that electrospinning does not impose a conductivity ceiling; the carrier does, and the carrier can be removed. Chemical polymerization retains a genuine advantage in that doping is built into the synthesis and requires no additional step.

Post-treatment is consistently the largest single lever. Thermal annealing followed by hot sulfuric acid raises PAN/PEDOT:PSS mats to 3.2 × 10^−3^ S/cm from the 10^−6^ to 10^−4^ S/cm of untreated PEO- and PVA-based analogues [67], polar-solvent treatment with dimethyl sulfoxide or ethylene glycol raises PEDOT:PSS fiber conductivity by orders of magnitude by removing insulating PSS and promoting chain ordering [35,66], and vapor-phase conversion of PLA fibers reaches 1.5 × 10^−1^ S/cm [68]. The practical conclusion is that electrospinning does not impose a conductivity ceiling; the carrier does, and the carrier can be removed. Chemical polymerization retains a genuine advantage in that doping is built into the synthesis and requires no additional step, which matters for cost and for process simplicity even where it does not raise the attainable value.

### 5.7. Percolation and Network Connectivity

The conductivity difference between a blended electrospun mat and a conformally coated fiber is not only a matter of how much conductive polymer is present. The two architectures reach conductivity by different connectivity mechanisms, and recognizing this explains several of the numbers in Table 2 that otherwise look anomalous.

In a blended mat, the conductive polymer is dispersed as a minority phase inside an insulating carrier. Charge transport requires a continuous path through the conducting phase, which exists only above a percolation threshold, the loading at which dispersed conducting domains first form a connected network spanning the fiber. Above that threshold, conductivity follows a power law in the excess loading, so small changes in composition near the threshold produce large changes in conductivity, while far above it, additional loading has little effect [101]. Anisotropic domains percolate at much lower loadings than spherical ones because elongated objects overlap more readily, which is why fibrillar PANI disperses more effectively than granular PANI at the same mass fraction [101]. A mat then imposes a second requirement in series, since current must also cross from fiber to fiber at physical contacts. Mat conductivity is therefore limited by whichever of the two connectivity requirements is less well satisfied.

A conformally coated fiber does not require percolation within the fiber, because the conductive polymer forms a continuous shell by construction. Two requirements remain. The shell itself must be continuous along the fiber, which depends on the uniformity of the oxidant loading and of the vapor exposure, and in a mat the current must still cross from fiber to fiber at physical contacts. Coating therefore removes one of three connectivity requirements rather than all of them, which is why a coated mat still reads below a single coated filament. This is the physical reason that post-spinning conversion recovers much of the difference relative to blending, as reported in Section 5.6, and it explains why carrier removal is so effective, since removing the insulating phase converts a percolating composite into a continuous conductor. The design implication is direct. Where high conductivity is required, the conducting phase should be continuous rather than dispersed, and electrospinning is best used to define the architecture on which that continuous phase is subsequently formed.

### 5.8. Process Scalability and Throughput

Conventional polymerization methods (e.g., bulk chemical oxidation or electrodeposition) can be scaled by increasing reactor or electrode size [102]. Bulk solution polymerization can produce large quantities of powder or coatings but requires thorough mixing, precise temperature control, and time-consuming purification steps. In practice, solution casting or printing of conductive polymers (such as PEDOT:PSS) has enabled roll-to-roll manufacturing of flexible films and devices [103]. For example, a high-resolution PEDOT:PSS ink was designed for roll-to-roll printing, achieving meter-scale patterning at room temperature [103]. By contrast, interfacial polymerization (at liquid to liquid or emulsified interfaces) inherently yields continuous films or fibers that can cover large areas directly [102,104]. Recent work showed that oil–water interface polymerization of pyrrole can produce kilometers of nanofiber material using simple stirring [102]. Although interfacial methods may be slower per unit area than rapid dip- or spin-coating, they bypass bulky waste handling and allow high overall throughput through parallel reactors or continuous flow. Notably, thermal CVD methods for polymers have low deposition rates (typically 1 to 10 nm/min [105]), whereas solution or interface routes cure in seconds or minutes over large areas, highlighting the throughput advantage of liquid-phase processing.

## 6. Applications for PANI-Based Conductive Fibers

Polyaniline is the most extensively applied conductive polymer in fiber form, and this section surveys five application areas in which both fabrication routes are represented. Although Section 5.2 showed that PEDOT:PSS outperforms PANI in raw conductivity, PANI has the deepest application literature because it is synthesized by a simple one step oxidative reaction and because its redox activity suits a wider range of transduction modes. Only the wearable sensing subsection bears directly on the third comparison criterion of this review. The remaining subsections are included as context for the breadth of the field and are not used as evidence for the route comparison.

### 6.1. Biosensing and Chemical Sensing

In biosensing, PANI fibers provide a high surface area for immobilizing enzymes or catalysts. Polymerization methods such as drop casting or in situ chemical growth coat electrodes with PANI, but the resulting morphology is set indirectly by reaction conditions rather than by a process parameter, so aggregate size and surface area vary between batches [39,43]. Electrospun PANI mats, by contrast, have a fiber diameter traceable to stated process parameters (Table 2), and it is this architecture that underlies the sensitivity improvements reported in the examples below. Kaewda and Sriwichai electrospun polyaniline and carbon nanotube composite nanofibers and obtained a label free dopamine biosensor with good sensitivity and operational stability [106], and an electrospun PEO/PEDOT:PSS nanofiber device was used to monitor both joint deformation and skin pH while retaining conductivity under bending [74]. Coaxial electrospinning extends this further by placing a functional phase in the shell rather than blending it into a single dope: a PAN/PANI core with a graphene oxide nanoscroll shell, subsequently loaded with gold nanoparticles, ferrocene and immobilized glucose oxidase, produced free-standing mats with approximately 40% porosity and a mean fiber diameter near 200 nm for sweat glucose sensing [87]. Comparing the polymers themselves rather than the routes, PEDOT:PSS generally has higher intrinsic conductivity and stability than PANI, while PANI is cheaper and easier to synthesize in bulk [91]. In each of these cases, the nanoscale fiber architecture raises the accessible electrode area and shortens the diffusion path to the immobilized recognition element [42,106].

### 6.2. Supercapacitors and Energy Storage

**Figure 8:**
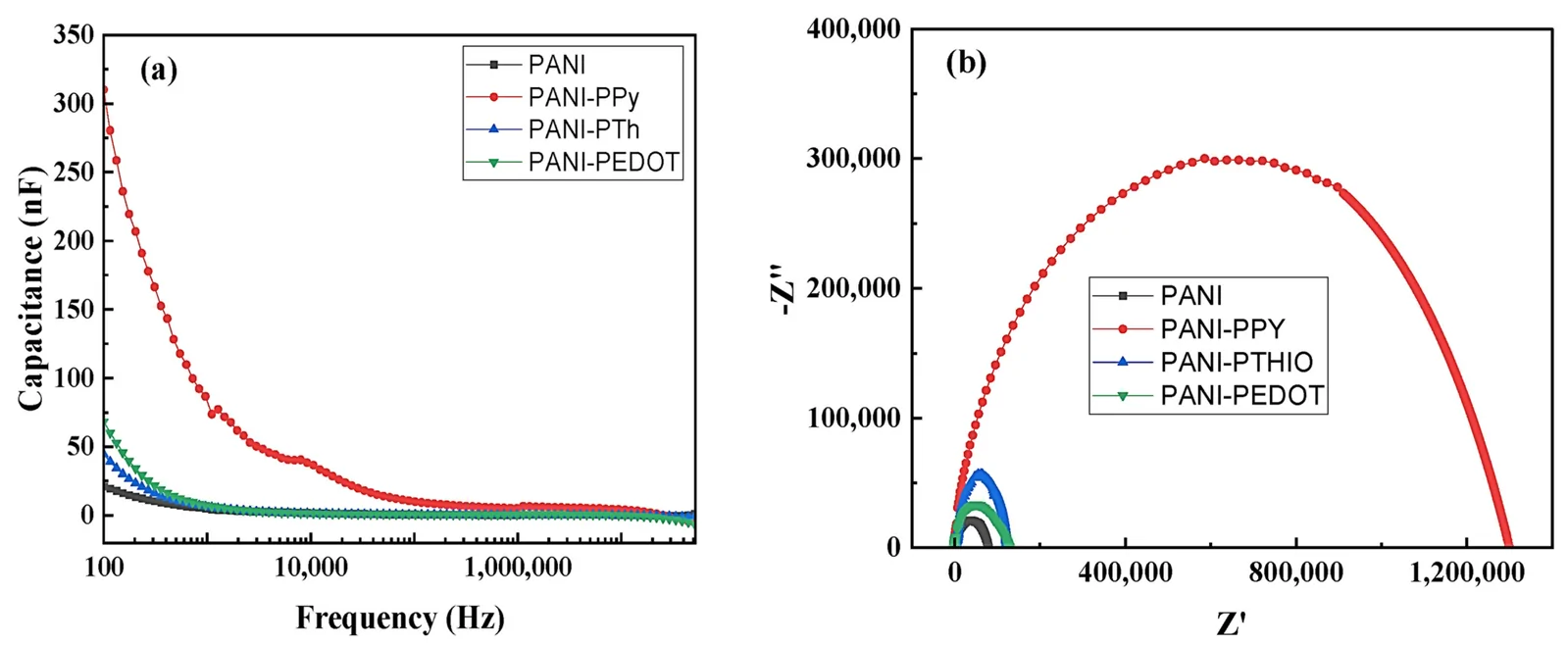
Supercapacitor performance of PANI and PANI copolymer electrodes. (**a**) Frequency dependent specific capacitance and (**b**) Cole-Cole (Nyquist) plots for pure PANI and various PANI copolymers. The PANI-PPy electrode shows the highest capacitance in (**a**) and, in (**b**), a single depressed semicircle substantially larger than those of the other copolymers, indicating a higher interfacial charge transfer resistance. Reproduced from [108] under CC BY 4.0.

For supercapacitors and batteries, PANI nanofibers are valued for pseudo capacitance. Chemically polymerized PANI can be grown on carbon cloth or templates to boost electrode capacity, as, for example, PANI-coated carbon nanofiber composites achieved approximately 157 F/g capacitance [107]. Electrospinning offers an alternative: binder-free PANI nanofiber mats (often with CNTs or carbon) have yielded even higher capacitances. One study reported approximately 308 to 385 F/g for pure PANI and PANI–CNT electrospun electrodes [73], with approximately 70 to 81% retention after 1000 cycles. This performance stems from the mats’ interconnected porous architecture that ensures efficient electron pathways and ion diffusion [73]. Copolymers also show intriguing effects: combining PANI with PPy or PEDOT modifies stability and capacitance. Kawamura et al. found PEDOT:PSS films to have higher conductivity than PANI [91], but PANI-PPy copolymer electrodes exhibited the highest specific capacitance approximately 420 F/g with 97.8% retention among PANI composites [108]. Figure 8 illustrates this: the PANI-PPy electrode retains far greater capacitance across the measured frequency window than pure PANI or PANI-PTh (8a), and in the Cole-Cole representation (8b) it traces a single depressed semicircle roughly six times larger along the real axis than those of PANI, PANI-PTh and PANI-PEDOT, which all close within a narrow region near the origin [108]. All four spectra begin at a small real axis intercept, indicating comparable electrolyte and contact resistance. The diameter of the semicircle corresponds to the resistance of the interfacial charge transfer and redox process, so the larger arc indicates that the higher capacitance of PANI-PPy is accompanied by slower interfacial kinetics rather than faster ones, and the original authors likewise report that the impedance of PANI-PPy exceeds that of the other electrodes [108]. The capacitance advantage of 420 F/g at 10 mV/s, with 97.8% retention after 100 cycles, therefore, comes with a rate penalty that matters for a wearable device operated at high current density. The performance advantage of electrospun electrodes in energy storage is therefore attributable to controlled pore structure and high electroactive area, both of which follow from the reproducibility of fiber diameter demonstrated in Section 5.5. Chemically polymerized PANI can reach comparable capacitance when grown on a pre-formed porous scaffold, but the scaffold geometry is then doing the same work that electrospinning does directly [73].

### 6.3. Wearable and Flexible Electronics

Table 3 groups the surveyed devices by transduction mode, because the metrics are not mutually convertible and no single scale ranks them. Within each mode the devices differ not only in fabrication route but also in the conductive phase added, whether silver nanowires, carbon nanotubes or a catalyst, and in the substrate that carries them. No surveyed study varies the fabrication route while holding those factors constant, so the reported performance cannot be attributed to the route. What the table supports is narrower: fibrous and nanostructured architectures provide the accessible surface area on which these transduction mechanisms depend, and both routes produce such architectures. An electrospun polyurethane nanofiber membrane carrying a silver nanowire and PANI dual network reports the highest gauge factor in the set [109], and a nanostructured PANI composite on a flexible electrode the lowest gas detection limit [110], but both carry an added conductive phase whose contribution is not separable from that of the architecture. In situ polymerization on nylon or cotton remains the most direct way to obtain a conductive fabric and gives sheet resistances of 128 to 724 Ω/sq [111], a quantity that is not comparable with the others for the reasons set out in Section 4.1.

The metrics used in Table 3 are defined as follows. Gauge factor is the fractional change in resistance divided by the applied strain and is meaningful only together with the strain range over which it was measured. Pressure sensitivity is the fractional change in resistance or capacitance per unit applied pressure, in kPa^−1^, and is not convertible to a gauge factor. Limit of detection is the lowest analyte concentration giving a response distinguishable from the baseline. Response and recovery times are the intervals taken to reach and to return from a stated fraction of the steady state response, conventionally 90%. Hysteresis is the difference between the loading and unloading response at the same stimulus, as a percentage of full scale. Cycling stability is the response retained after a stated number of cycles. Washing durability is the response retained after a stated number of laundering cycles under a named protocol. Skin to electrode contact impedance is quoted at a stated frequency. These quantities are not mutually convertible, which is why Table 3 groups devices by transduction mode rather than ranking them on a single scale.

A wearable device must satisfy four mechanical requirements in addition to its transduction performance: it must flex without loss of continuity, stretch with the skin or the garment, survive repeated bending, and survive laundering. None of the devices compiled in Table 3 reports all four. Bending endurance is reported for a minority and washing durability for none, and the two durability studies identified in this survey concern coated textiles that are not among the devices compared here [75,76]. This is the principal gap between the surveyed laboratory results and a deployable device, and it applies equally to both fabrication routes.

Pressure and tactile sensing is a distinct transduction mode that deserves separate treatment, because the sensing mechanism differs from that of strain devices. A strain sensor responds to in plane elongation, which separates conducting pathways and raises resistance. A pressure sensor responds to through thickness compression, which brings conducting elements closer together and lowers resistance, and its sensitivity therefore depends on the compressibility of the sensing layer rather than on its extensibility. Porous electrospun mats are well suited to this mode because their high void fraction allows large compressive deformation at low applied pressure, and because the number of fiber-to-fiber contacts increases steeply with compression. Conductive polymer coated foams and mats have accordingly been used for tactile and pressure devices, with the porosity of the fibrous network providing the mechanical compliance and the conducting coating providing the electrical response [5].

### 6.4. Biopotential and Electrophysiological Electrodes

Recording electrocardiography, electromyography and other biopotentials requires an electrode that couples ionic current in tissue to electronic current in an instrument. The clinical standard is a silver chloride electrode with a wet gel, which performs well but dries out, irritates skin over long wear and cannot be integrated into a garment. Conductive polymers are attractive replacements because they conduct both ionically and electronically, which lowers the impedance of the tissue to electrode interface, and because they are soft enough to conform to skin.

The governing metric is skin-to-electrode contact impedance, usually quoted at frequencies relevant to the signal of interest, and lower impedance means less noise and fewer motion artifacts. Fully textile electrodes made by soaking fabric in PEDOT:PSS with a secondary dopant have been tested on human volunteers at rest and during exercise, and in wet operation they gave stable and reproducible behavior comparable to or better than disposable gelled silver chloride electrodes, while dry operation proved more prone to motion artifacts because skin contact was less stable [113]. Improving that contact is the main line of current work. An intrinsically conductive, stretchable and self-adhesive PEDOT:PSS based dry electrode has been shown to maintain conformal skin contact during movement and sweating, delivering long term motion robust recordings without gel [114].

For the comparison of this review, the relevant point is that this application depends on conformability and interfacial area rather than on bulk conductivity. Electrospun mats offer high interfacial area and mechanical compliance, and in situ coating offers direct integration into an existing garment, so neither route is excluded on principle. What limits progress is the same durability gap identified elsewhere in this review, since electrode impedance after repeated wear and washing is rarely reported.

### 6.5. Actuators, Filtration and EMI Shielding

#### Environmental and Operational Stability

Five environmental factors govern how long a conductive polymer fiber device remains usable. The surveyed literature treats them separately, which is why they are collected here. Humidity acts at two stages. During fabrication, it governs charge dissipation from the electrospinning jet and the rate of solvent evaporation and therefore influences almost every property of the collected fiber [77]. In service, it promotes dedoping of acid doped PANI, whose conductivity falls as the dopant is lost, and even high-performance films degrade gradually under sustained humidity or strain [91,92]. Thermal cycling drives the same loss and additionally relaxes the chain ordering produced by post-treatment. Oxidation of the polymer backbone is a slower and irreversible pathway, and it is distinct from dedoping, which can be reversed by redoing. Sweat exposure combines the two, since sweat is an aqueous electrolyte that both leaches dopant and wets the fiber network, and it is the operating environment assumed by the sweat sensors discussed in Section 6.1 and Section 6.3. Biocompatibility is a separate requirement for skin-mounted devices and is not established by the electrical and mechanical measurements surveyed here. Across the surveyed set, these factors are reported inconsistently and without a shared protocol, and standardized wash testing appears in a minority of studies [75,76], so no quantitative comparison of the two fabrication routes on stability is possible from the present literature.

PANI-based materials have also found use in electrochemical actuators and environmental applications. In actuation, PANI’s volumetric changes under redox stimuli can produce motion: for instance, a PANI/CNT yarn artificial muscle generated a large contractile stroke approximately 17% and high isometric stress (approximately 8 MPa) at voltages < 2 V in aqueous electrolyte [115]. This performance (stroke and stress) surpasses many prior conducting-polymer actuators, making such composites promising for soft robotics or haptic devices. For filtration, PANI is often used in membranes and adsorbents: e.g., PANI-coated polyimide (PI) membranes achieved >99% rejection of a model dye (Congo red) with only minor flux loss [116], and PANI polyether sulfone nanofiltration membranes filtered 43% of NaCl at 1.7 bar (flux approximately 11.6 L·h^−1^·m^−2^) [117]. These results highlight PANI’s utility in water purification (removing organics or salts) and air filters (adsorbing particulates or gases) driven by its charge adsorption and redox behavior.

Thicker PANI composites absorb and reflect microwaves more effectively than thin coated fabrics. Thin PANI-coated fabrics of approximately 0.1 mm give moderate shielding (11 to 15 dB) across 8 to 18 GHz [25]; the shielding effectiveness scales with coating thickness and PANI loading, making bulk composites better shields at the cost of flexibility. Free-standing PANI/poly(vinyl butyral) films (0.78 mm) reached approximately 26 dB in the X-band and approximately 30 dB in the Ku-band [118], while PANI/graphene nanoflake films of approximately 10 µm reached approximately 57 dB across the S/X-bands [118].

## 7. Conclusions

Conductive polymer fibers are made by two routes that differ less in the properties they can ultimately reach than in where control over the fiber is exerted. Chemical oxidative polymerization grows the polymer into whatever morphology the reaction conditions produce, so geometry follows indirectly from monomer concentration, acidity, oxidant ratio and mixing regime. Electrospinning imposes geometry through a dedicated fiber-forming step in which diameter, alignment and porosity are set by parameters the operator controls. Judged on the three criteria used here, that difference resolves in favor of electrospinning for two of them and leaves the third with chemistry.

On reproducibility, the evidence is unambiguous. Every surveyed electrospun system specifies fiber diameter as a numeric range traceable to stated process parameters, while chemically polymerized systems describe their products qualitatively. The one chemical route that reports a controlled dimension, wet spinning, is also the only one in which the fiber is formed by extrusion and drawing rather than by precipitation, and it is the same route that produces the highest mechanical performance in the surveyed set. Reproducible geometry therefore tracks the presence of a spinning operation rather than the choice of chemistry.

On electrical performance, the comparison has been distorted by measurement geometry. Single-fiber, mat, pellet and sheet-resistance values differ by one to three orders of magnitude for the same material and are nonetheless quoted interchangeably. Once geometry is held constant, the deficit of electrospun fibers is attributable to dilution by the carrier polymer rather than to the spinning process, and carrier removal, post-treatment or post-spinning conversion recover two to four orders of magnitude, bringing electrospun material into the range of chemically derived bulk. In wearable sensing, the spun and post-converted architectures deliver the higher reported sensitivities, because the accessible surface area on which those devices depend is a direct consequence of controlled fiber geometry.

Chemical polymerization retains one advantage that electrospinning has not matched: doping is built into the synthesis, so no additional conversion step is required. Throughput figures for the two routes are not directly comparable, because they are measured at different endpoints. Bulk oxidative polymerization is reported in kilograms of polymer powder, before purification, drying and any subsequent shaping step. Laboratory electrospinning is reported in tens of milligrams per hour, and roll-to-roll systems in hundreds of grams per hour, of finished fiber mat [46,47,52]. Compared at the common endpoint of finished conductive fiber ready for device integration, the chemical route retains an advantage in quantity, but the size of that advantage cannot be stated from the present literature, because the intermediate steps are not reported with their yields. The constructive reading is not that one route supersedes the other but that their roles are separable. Chemistry is the better supplier of the conducting phase; a spinning process is the better supplier of the architecture.

Three limits of this comparison should be stated plainly. The surveyed studies differ simultaneously in composition, doping, architecture, processing and testing conditions, so differences between them cannot, in general, be attributed to the fabrication route; only three paired comparisons were identified in which a single study varies one factor at a time. No replicate batch data and no inter-laboratory comparison were found for either route, so neither can be said to be the more reproducible. The wearable devices surveyed here are characterized on metrics that are not mutually convertible, so no ranking of the routes on sensing performance is possible from the present literature. What the evidence does support is that the two routes operate at different stages of fabrication and are complementary, and that comparability in this field is limited less by the materials than by what is reported about them.

Three obstacles should shape the next phase of work. Throughput is the clearest, and multi-jet, needleless and centrifugal configurations need to be assessed on fiber quality rather than on rate alone [46,47,52]. Long-term stability is the least well documented, since dedoping under humidity, thermal cycling and repeated deformation is reported inconsistently and standardized protocols such as ISO 6330 wash testing appear in a minority of studies [75,76]. Solvent use is the third, as electrospinning of CP systems still relies on chloroform, dimethylformamide and m-cresol, so the aqueous and green-solvent chemistry already demonstrated for interfacial polymerization deserves systematic extension to spinning [41]. Above all, the field would benefit from reporting fiber diameter, conductivity with its measurement geometry, mechanical properties and stability on the same specimens, which would allow the routes to be compared quantitatively rather than by assertion.

A further route to closing that gap is computational rather than experimental. The relation between electrospinning parameters and fiber diameter is nonlinear and involves several interacting variables, which is precisely the situation in which data-driven models outperform intuition. Predictive models trained in diameters compiled from published work have reached coefficients of determination above 0.9 for fiber diameter prediction, and sensitivity analysis within those models consistently identifies polymer concentration, applied voltage and feed rate as the dominant variables [119]. Because such models are built from literature data, they also make the reporting problem visible, since a study that omits humidity or solution age cannot contribute to a usable record.

Extending this approach to conductive polymer systems specifically requires datasets that pair process parameters with conductivity and its measurement geometry, which do not presently exist in the required form. Assembling them is a realistic community objective and would deliver two things at once. It would give a predictive process-to-structure–property model of the kind that the field currently lacks, and it would enforce, as a condition of contributing data, exactly the reporting discipline that this review argues is missing.

## 8. Future Work

Three lines of work would move this field from qualitative comparison toward quantitative prediction. The first is a coordinated inter-laboratory study. No round robin exercise for conductive polymer fibers was identified in this survey, so the most immediate contribution available to the community is a shared protocol executed in several laboratories, reporting fiber diameter distributions with coefficients of variation and conductivity with its measurement geometry stated explicitly. Such a study would establish for the first time whether published protocols transfer between groups, which is the question that reproducibility claims ultimately rest on. The second is systematic durability characterization. Conductivity before and after mechanical cycling, humidity exposure and standardized laundering is reported for only a minority of devices, and almost never on the same specimens used for the initial electrical measurements, so the durability advantage frequently claimed for post-converted fibers over simple coatings remains untested. The third is the development of datasets suitable for predictive modeling. Existing machine learning work on electrospinning predicts fiber diameter from process parameters with useful accuracy, but no comparable dataset pairs process conditions with conductivity, measurement geometry and mechanical properties for conductive polymer systems. Assembling one would enable process-to-structure-to-property models for both routes and would, as a condition of contributing data, enforce precisely the reporting discipline this review argues is currently absent. Alongside these, greener solvent systems for electrospinning and higher throughput configurations such as needleless and multi-jet spinning deserve evaluation on fiber quality rather than on production rate alone.

## Figures and Tables

**Table 1:** Qualitative comparison of chemical oxidative polymerization and electrospinning as routes to conductive polymer fibers. Entries are summary statements supported by the sections cited in the text and are not quantitative measurements.

Aspect	Chemical Polymerization	Electrospinning
Synthesis	Bulk oxidative polymerization of monomers (e.g., aniline + oxidant in acid)—a simple, low-cost batch process. No special equipment beyond stirrer/heating [96].	Fiber spinning process: a polymer solution is ejected under high voltage. Requires specialized electrospinning setup (power supply, syringe pump, collector) and careful control of solution properties [98].
Materials Needed	Aniline monomer, oxidant (e.g., APS), acid, solvent. Materials are inexpensive and readily available. No polymer carriers needed [96].	PANI must be dissolved or dispersed: often requires a carrier polymer (PVP, PEO, etc.) and dopant (e.g., CSA) to form a spinnable solution. Uses volatile organic solvents [96,97].
Product Form	Typically yields powders, coatings or thick films. Fiber geometry is not set directly by the process and requires templating, interfacial reaction or a separate spinning step.	Produces nonwoven fiber mats (or aligned yarns). Fibers can be nanometers–microns in diameter, allowing tailored orientation or layering [98].
Morphology & Surface Area	Chemical oxidation usually yields micro-scale particles (often microns in size) with relatively low surface area. Fiber structure is not directly obtained [99].	Electrospun fibers have nanofibrous morphology: small diameters, high porosity, and very large surface-to-volume ratio. This increases active surface area dramatically [98].
Electrical Conductivity	High in dense form. Pressed PANI pellets can reach conductivities on the order of 2–21 S/cm (depending on doping level and compaction) [96].	Geometry-dependent. As-spun mats of carrier-diluted blends: 10^−6^ to 10^−2^ S/cm. Single-fiber values for neat or post-converted systems: 10^0^ to 10^1^ S/cm. post-spinning doping or carrier removal is consistently the largest lever (Section 4.1) [96].
Production Rate & Scalability	High yield per batch; easily scaled by increasing batch size. Does not require specialized machinery.	Low throughput: Limited by single-needle flow rates. Scaling up requires parallel setups or specialized multi-jet systems. Requires skilled operation and is less mature industrially [100].
Advantages	Cost-effective: simple reagents/equipment - High yield: easily produces bulk quantities of PANI - Stability: yields PANI with good environmental stability [96]	- Nano structuring: creates uniform, continuous nanofibers with tunable diameter and orientation—Surface area: fiber mats have very large interfacial area (good for sensors, catalysis) [98] - Flexibility: can incorporate complex morphologies (porous, core–shell, etc.) through process variations.
Limitations	- Indirect morphology control: fiber geometry follows from reaction conditions rather than from a process setting, so dimensions are rarely specified. - Moderate surface area: bulk PANI has less accessible surface than nanofibers (nanoscale PANI can improve conductivity) [99].	- Solubility issues: PANI’s poor solubility makes electrospinning difficult so it requires dopants or carrier polymers. - Lower as-spun conductivity: fibers often need post-treatment to approach bulk conductivity. - Equipment & throughput needs high-voltage setup so scale-up and reproducibility can be challenging [96,97].
Typical Applications	Bulk PANI (from chemical polymerization) is used in coatings, sensors, batteries, supercapacitors, LEDs, actuators, etc. Forms like films or pellets are common in electronic and energy devices [96].	Electrospun PANI fibers are explored for smart textiles, wearable sensors, filtration membranes, gas sensors, and catalysis. High-porosity fiber mats enhance sensitivity and reaction rates [96,98]

**Table 2:** Compiled fiber dimensions and properties reported for conductive polymer fibers produced by chemical polymerization and by electrospinning. Conductivity values are as reported by the original authors and are not always measured by the same method, so they should be compared as orders of magnitude rather than as exact figures.

Material System	Route	Diameter	σ (S/cm)	Geometry	Other Reported Performance	Ref.
PANI, CSA-doped	Chemical, wet spinning	<5 µm	n.r.	—	1080 MPa tensile strength; 30 GPa modulus; >5 km spun in 2 h	[33]
PANI	Chemical, interfacial (oil–water)	nanofibrous	n.r.	—	green route; symmetric supercapacitor electrodes	[41]
PANI	Chemical, bulk (rapid mixing)	thin, interconnected	n.r.	—	≈1000 mF/cm^2^ areal capacitance, double that of stirred PANI	[44]
PPy on aramid nanofiber/CNT aerogel	Chemical, in situ on fiber	aerogel fiber	6.4 × 10^−2^	fiber	56 mg/cm^3^; strain and temperature sensing	[45]
PANI on cotton and nylon fabric	Chemical, in situ on textile	nanofiber coating	n.r.	—	11–15 dB EMI shielding, 8–18 GHz, at 100 µm	[25]
PANI/PEO	Electrospinning, blend	0.9–1.9 µm	rises with PANI content	mat	below equivalent cast film	[58]
PANI/PS	Electrospinning, blend	72–100 nm	rises with PANI content	mat	much finer fibers than PANI/PEO	[59]
PEDOT:PSS + 0.5 wt% high-MW PEO	Electrospinning, blend	300–400 nm	≈35	single fiber	smooth, bead-free fibers	[4,60]
PANI core/PMMA sacrificial shell	Electrospinning, coaxial	≈620 nm	high after shell removal	fiber	neat CP fiber, no permanent carrier	[4]
PAN/PEDOT:PSS, 240 °C + hot H_2_SO_4_	Electrospinning + post-treatment	440–940 nm	3.2 × 10^−3^	mat	exceeds untreated PEO- and PVA-based analogues	[67]
PEO/CSA-doped PANI (Janus)	Electrospinning, side-by-side	submicron	10^−5^ to 10^−4^	mat	8–15× higher than the equivalent blend	[69]
PVP + ferric toluate, EDOT vapor	Electrospun template + VPP	≈350 nm (hollow)	≈60	mat	hollow PEDOT fibers after PVP removal	[4]
PEDOT on nitrile rubber/PEGDMA	Electrospun + in situ	submicron	≈6	mat	remains stretchable after coating	[4]
PEDOT on PLA	Electrospun + VPP	aligned fibers	1.5 × 10^−1^	mat	neural regeneration scaffold	[68]
PAN/PANI core, GONS shell	Electrospinning, coaxial	≈200 nm	0.048 to 0.060	mat	≈40% porosity; GOx immobilized; plastic deformation under compression	[87]

**Table 3:** Reported wearable-sensing performance for conductive polymer fiber and coating devices. Metrics are not mutually convertible and are grouped by transduction mode; sheet resistance is included where the source reports no sensitivity figure.

Device	Route	Reported Metric	Ref.
PANI/AgNW on polyurethane nanofiber membrane	Electrospun + coating	strain sensor, gauge factor ≈ 59 over 0–35% strain	[109]
PEO/PEDOT:PSS nanofiber flex sensor	Electrospun blend	physiological flex sensing; conductivity retained on bending	[74]
PANI/CNT nanofiber electrode	Electrospun blend	label-free dopamine detection	[106]
Pt/MoS_2_/PANI on polyimide	Chemical, nanostructured composite	NH_3_ gas sensor, ≈250 ppb detection limit at room temperature	[110]
PANI on PET substrate	Chemical, in situ	sweat pH sensor, ≈62.3 mV/pH, near-Nernstian	[112]
PANI on nylon fabric	Chemical, layer-by-layer	conductive textile electrode, 128–724 Ω/sq sheet resistance	[111]
PEDOT:PSS on cotton textile	Chemical, in situ coating	ECG dry electrode; contact impedance comparable to gelled Ag/AgCl	[113]
Self-adhesive stretchable PEDOT:PSS film	Chemical, solution processed	motion-robust ECG and EMG recording without gel	[114]

## Data Availability

No new data were created or analyzed in this study. Data sharing is not applicable to this article.

## Abbreviations

The following abbreviations are used in this manuscript:

AFM	Atomic Force Microscopy
AgNW	Silver Nanowire
APS	Ammonium Persulfate
BET	Brunauer–Emmett–Teller
CNT	Carbon Nanotube
CPs	Conductive Polymers
CSA	Camphor sulfonic Acid
CV	Cyclic Voltammetry
CVD	Chemical Vapor Deposition
DMSO	Dimethyl Sulfoxide
DMF	Dimethylformamide
dB	Decibel
EDOT	3,4-ethylenedioxythiophene
EIS	Electrochemical Impedance Spectroscopy
EMI	Electromagnetic Interference
ES	Electrospinning
F/g	Farads per gram
FeCl_3_	Ferric Chloride
GF	Gauge Factor
GHz	Gigahertz
GPa	Gigapascal
HCl	Hydrochloric Acid
H_2_SO_4_	Sulfuric Acid
ICP	Intrinsically Conductive Polymer
MO	Methyl Orange
MPa	Megapascal
MW	Molecular Weight
mV	Millivolt
Ω/sq	Ohms per square
PAN	Polyacrylonitrile
PANI	Polyaniline
PCL	Polycaprolactone
PEDOT	Poly(3,4-ethylenedioxythiophene)
PEDOT:PSS	Poly(3,4-ethylenedioxythiophene):Polystyrene sulfonate
PEO	Poly(ethylene oxide)
PET	Poly(ethylene terephthalate)
PLA	Polylactic acid
PMMA	Poly(methyl methacrylate)
ppb	Parts per billion
ppm	Parts per million
PS	Polystyrene
PSS	Polystyrene sulfonate
PPy	Polypyrrole
PU	Polyurethane
PVA	Poly(vinyl alcohol)
PVP	Poly(vinylpyrrolidone)
S/cm	Siemens per centimeter
SEM	Scanning Electron Microscopy
TEM	Transmission Electron Microscopy
TIP	Template-assisted Interfacial Polymerization
VPP	Vapor-Phase Polymerization
XRD	X-ray Diffraction
e-textiles	Electronic Textiles
pH	Potential of Hydrogen
